# MAPK-dependent control of mitotic progression in *S. pombe*

**DOI:** 10.1186/s12915-024-01865-6

**Published:** 2024-03-25

**Authors:** Ana Belén Iglesias-Romero, Terersa Soto, Ignacio Flor-Parra, Silvia Salas-Pino, Gabriel Ruiz-Romero, Kathleen L. Gould, José Cansado, Rafael R. Daga

**Affiliations:** 1 Centro Andaluz de Biología del Desarrollo, Universidad Pablo de Olavide, Seville, 41013 Spain; 2 Yeast Physiology Group, Department of Genetics and Microbiology, Facultad de Biología, Universidad de Murcia, Murcia, 30071 Spain; 3 Department of Cell and Developmental Biology, Vanderbilt University School of Medicine, Nashville, TN 37240 USA

**Keywords:** MAPK, CIP, SAPK, Pmk1, Sty1, Spindle assembly checkpoint, MCC, CDC20/Slp1, APC/C, Osmotic stress, Mitosis, Cytokinesis

## Abstract

**Background:**

Mitogen-activated protein kinases (MAPKs) preserve cell homeostasis by transducing physicochemical fluctuations of the environment into multiple adaptive responses. These responses involve transcriptional rewiring and the regulation of cell cycle transitions, among others. However, how stress conditions impinge mitotic progression is largely unknown. The mitotic checkpoint is a surveillance mechanism that inhibits mitotic exit in situations of defective chromosome capture, thus preventing the generation of aneuploidies. In this study, we investigate the role of MAPK Pmk1 in the regulation of mitotic exit upon stress.

**Results:**

We show that *Schizosaccharomyces pombe* cells lacking Pmk1, the MAP kinase effector of the cell integrity pathway (CIP), are hypersensitive to microtubule damage and defective in maintaining a metaphase arrest. Epistasis analysis suggests that Pmk1 is involved in maintaining spindle assembly checkpoint (SAC) signaling, and its deletion is additive to the lack of core SAC components such as Mad2 and Mad3. Strikingly, *pmk1Δ* cells show up to twofold increased levels of the anaphase-promoting complex (APC/C) activator Cdc20^Slp1^ during unperturbed growth. We demonstrate that Pmk1 physically interacts with Cdc20^Slp1^ N-terminus through a canonical MAPK docking site. Most important, the Cdc20^Slp1^ pool is rapidly degraded in stressed cells undergoing mitosis through a mechanism that requires MAPK activity, Mad3, and the proteasome, thus resulting in a delayed mitotic exit.

**Conclusions:**

Our data reveal a novel function of MAPK in preventing mitotic exit and activation of cytokinesis in response to stress. The regulation of Cdc20^Slp1^ turnover by MAPK Pmk1 provides a key mechanism by which the timing of mitotic exit can be adjusted relative to environmental conditions.

**Supplementary Information:**

The online version contains supplementary material available at 10.1186/s12915-024-01865-6.

## Background

The mitotic checkpoint or spindle assembly checkpoint (SAC), is a surveillance mechanism that contributes to the preservation of genome stability by delaying the timing of mitotic exit until all chromosome are properly attached to the mitotic spindle [1–3]. A defective SAC leads to aneuploidy and results in embryonic lethality, premature aging in mouse models and correlates with tumor malignancy [4–7].

Kinetochores not only provide a structural platform for chromosomes to be captured by the spindle MTs, but also are where the checkpoint signal emanates from [8]. At mitotic onset, the master SAC regulator Mps1 kinase is recruited to kinetochores by Aurora B [9, 10]. Kinetochore-bound Mps1 (Mph1 in *S. pombe*) phosphorylates Kln1 (Spc7 in *S. pombe*) that acts in turn as a hub for binding to the SAC signaling components Bub1, Bub3, the Mad1-Mad2 complex, and BubR1 (Mad3 in *S. pombe*) [11–15]. Mad2 can adopt two different conformations: inactive Open Mad2 (O-Mad2) and an active Closed Mad2 (C-Mad2). The Mad1-C-Mad2 complex induces a conformational change of soluble 0-Mad2 at unattached kinetochores to form C-Mad2, which then binds CDC20 (Slp1 in *S. pombe*, Cdc20^Slp1^), Bub3, and BubR1, to assemble the mitotic checkpoint complex (MCC) that acts as diffusible anaphase-wait signal [16–21]. CDC20 is the activator and substrate adaptor of the E3 Ubiquitin ligase anaphase-promoting complex or cyclosome (APC/C). APC/C-Cdc20 ubiquitinates and marks for proteasomal degradation mitotic regulators such as securin and cyclin B, leading to chromosome segregation and exit from mitosis, respectively [22–25]. Upon checkpoint activation, CDC20-MCC binds APC/C-CDC20 which contributes to CDC20 turnover and APC/C inactivation. This avoids degradation of its mitotic substrates and delays mitotic progression [26–32]. Therefore, CDC20 functions as activator (APC/C-CDC20) and inhibitor (CDC20-MCC) of APC/C and mitotic progression. APC/C-CDC20 activity is regulated at multiple levels [reviewed in [33]]. Degradation of CDC20 contributes to maintaining proper levels of this key APC/C activator during mitotic arrest, thus increasing the robustness of the mitotic checkpoint [27, 34]. APC/C-CDC20 binding is also regulated through phosphorylation by Cdk1-cyclin B complexes. Phosphorylation of APC/C subunits promotes the association with CDC20 [ reviewed in [35]], whereas phosphorylation of CDC20 inhibits its binding to APC/C [36–38].

**Fig. 1:**
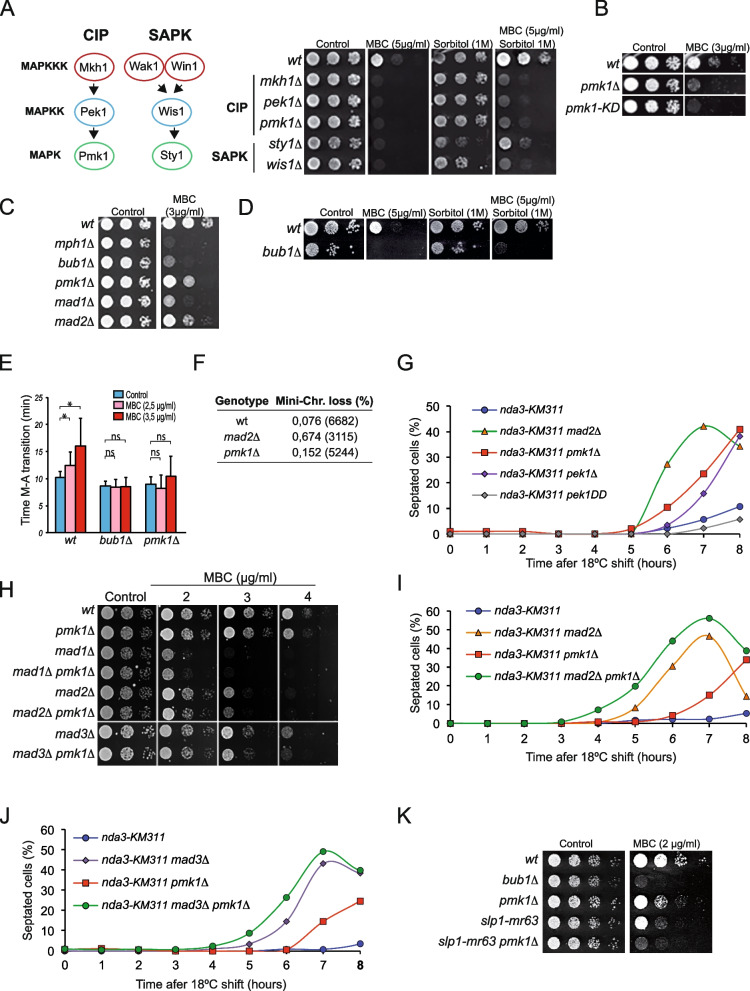
*pmk1Δ *cells display increased sensitivity to MBC and defective mitotic arrest. **A **Left: Schematic representation of the CIP and SAPK MAPK modules. Right: Spot growth assay of the indicated strains and conditions. **B–D** Spot growth assay of the indicated strains and conditions. Representative example of three independent experiments. **E** Average times from SPB separation until metaphase to anaphase transition is indicated for each strain. Sid2-GFP was used as an SPB marker.  Error bars indicate the standard deviation (SD) of two independent repetitions (*n*=30 cells). The asterisk denotes statistically significant differences (*p* < 0.05) and n.s.: not significant (*p* > 0.05). **F **Rate of minichromosome loss per cell division of the indicated strains. Total number of colonies counted from two independent experiments are in parenthesis**. G** Representative example of mitotic arrest assay of the indicated strains in the *nda3-KM311* background. Percentage of septation is shown as readout of mitotic exit/leakage; *n*=200 cells was quantitated for each strain and time point from two independent experiments. **H** Spot growth assay of the indicated strains in the presence of increasing concentration of MBC. Representative example of three independent experiments. **I** Representative example of mitotic arrest assay of the indicated strains. To gain further synchrony cells were pre synchronized with HU for 3h at 30°C, and then released at restrictive temperature (18°C) after HU washout. Percentage of septation is shown as readout of mitotic exit/leakage; *n*=200 cells were quantitated for each strain and time point from two independent experiments. **J **Representative example of mitotic arrest assay of the indicated strains. Percentage of septation is shown as readout of mitotic exit/leakage; *n*=200 cells were quantitated for each strain and time point from two independent experiments. **K** Spot assay of the indicated strains and conditions. Plates were incubated at 25°C. Representative example of three independent experiments

Mitogen-activated protein kinases (MAPKs) are evolutionary conserved signaling cascades composed of a three-tier kinase module that transduces extracellular stimuli into a variety of adaptive cellular responses [reviewed in [39]]. Fission yeast *S. pombe* has three MAPK cascades, two of which, the stress-activated protein kinase pathway (SAPK) and the cell integrity pathway (CIP), are analogous to the mammalian p38/JUNK and ERK pathways, respectively (Fig. 1A) [40]. Sty1 and Pmk1 are the respective MAPKs of the SAPK and CIP pathways and become activated by changes in physiological conditions or physicochemical perturbations including osmotic stress, cell wall damage, thermal stress, oxidative stress, and glucose limitation, among others [41–43]. MAPKs phosphorylate a myriad of cytoplasmic substrates as well as nuclear transcription factors that promote a concerted response that helps to maintain cellular homeostasis under adverse conditions [39, 43–45]. MAPKs have long been known as regulators of the spindle assembly checkpoint and APC/C activity in Xenopus eggs and tad pole cells [45–50]. These studies showed that MAPKs are transiently activated during mitosis and their activity is required for normal mitotic progression and for SAC establishment and maintenance in response to microtubule (MT) damage. However, the molecular mechanism and targets of MAPKs in mitotic regulation are largely unknown. In this work, we show that the *S. pombe* MAPK Pmk1 regulates Cdc20^Slp1^ turnover which is critical for proper mitotic checkpoint function and the response to stress.

## Results

### Inactivation of the CIP MAPK pathway causes hypersensitivity to MT damage

Carbendazim (MBC) is commonly used in yeasts to induce depolymerization of MTs, thus compromising the formation of the mitotic spindle and the ensuing process of chromosome capture [51]. This engages the spindle assembly checkpoint (SAC), which transiently delays exit from mitosis in such conditions. Continuous growth in the presence of MBC results in lethal mitosis and loss of cell viability [51, 52]. However, we unexpectedly found that sorbitol suppressed MBC-induced death of wild type *S. pombe* cells (Fig. 1A). Sorbitol is a non-fermentable sugar that produces intracellular water imbalance and elicits a rapid and strong osmotic stress response [41]. Growth in the presence of sorbitol activates both Pmk1 and Sty1, the respective MAPKs of the SAPK and CIP pathways [41, 53]. We found that deletion of Pmk1, Sty1, and the corresponding MAPK module components Mkh1 (CIP MAPKKK), Pek1 (CIP MAPKK), and Wis1 (SAPK MAPKK), resulted in MBC hypersensitivity (Fig. 1A). However, contrary to wild type cells, this phenotype was not suppressed in media supplemented with 1 M sorbitol (Fig. 1A), suggesting that MAPK pathways are required for this response. Since the sorbitol-recovery phenotype was more penetrant in the CIP mutants (Fig. 1A), we focused on characterizing the role of the CIP/Pmk1 in suppression of growth sensitivity to MBC by sorbitol.

The MBC sensitivity of a mutant bearing a catalytically inactive allele of *pmk1 (pmk1-KD, Kinase Dead)* [54] was similar to that of *pmk1Δ* cells (Fig. 1B), suggesting that catalytically active MAPK is necessary for cellular adaptation to MT depolymerization. The growth sensitivity of *pmk1Δ* cells to MBC was milder than that elicited by lack of the core SAC gene components Bub1, Mph1, or Mad1, but similar to that of *mad2∆* cells (Fig. 1C). Analogous to *pmk1Δ* cells, the suppression of MBC sensitivity by sorbitol was also lost in the absence of the SAC regulator Bub1 (Fig. 1D).

To further explore the biological relevance of the suppression of MBC sensitivity by sorbitol, we compared the rates of mitotic progression in wild type and *pmk1Δ* cells in the presence of increasing concentrations of this drug by measuring the distance between the spindle pole bodies (SPBs) using time-lapse microscopy. As expected, anaphase onset was extended in wild type cells proportionally to the increase in MBC concentration (Fig. 1E). In contrast, similar to *bub1Δ* cells [55], *pmk1Δ* cells failed to delay mitotic progression in the presence of MBC (Fig. 1E). We next comparatively checked the rate of minichromosome loss in wild type, *mad2Δ* and *pmk1Δ* strains. Consistently, *pmk1Δ* cells displayed a twofold increase in the rate of minichromosome loss per cell division compared to the wild type strain (Fig. 1F). These results suggest that the absence of Pmk1 causes defects in SAC signaling. Alternatively, since the CIP plays a role in cell wall maintenance [54], the growth sensitivity of *pmk1*Δ cells to MBC might be the result of increased drug intake due to a defect in the cell wall. To distinguish between these possibilities, we used a strategy in which MT dynamics is impaired by using the *nda3-KM311* allele that encodes a cold-sensitive mutant form of beta-tubulin [56, 57]. Under restrictive conditions (18–20 °C), *nda3* cells arrest mitotic progression in a SAC-dependent manner for several hours before leaking into cytokinesis. Therefore, the percentage of cells that proceed into cytokinesis and septation in this mutant is used as readout of decreased SAC functionality [58]. Whereas *nda3-KM311* cells started to leak from the mitotic arrest after approximately 8 h, the *nda3-KM311 pmk1*Δ double mutant escaped from the mitotic arrest at earlier times (5–6 h), and to a similar extent as *mad2Δ* cells, which are defective in mitotic checkpoint function (Fig. 1G). Deletion of the CIP MAPKK *pek1* (*pek1Δ*) phenocopied the behavior of *pmk1*Δ cells, whereas expression of a hyperactive *pek1* allele (*pek1-DD*), which constitutively maintains active CIP, slightly extended the metaphase arrest as compared to *nda3-KM311* alone (Fig. 1G). Together, the above results show that the CIP delays mitotic exit when spindle function is compromised.

### Pmk1 deficiency is additive to *mad2Δ*, *mad3Δ*, and *slp1-mr63*

Next, we explored the possible role of the CIP in SAC activation and/or maintenance by performing epistasis analysis between *pmk1*Δ cells and mutants deleted in the SAC components *mad1, mad2, or mad3.* The sensitivity to MBC of *pmk1*Δ cells was epistatic to deletion of *mad1* and slightly additive to deletion of *mad2 or mad3* (Fig. 1H). This phenotype was further investigated by using the *nda3-KM311* assay as above, but to gain further cell synchrony, cells were first treated with hydroxyurea (HU), which reversibly blocks cells in S-phase [59]. HU was washed out after 3 h and the cell cultures were then shifted to the restrictive temperature for the *nda3-KM311* allele. Under these conditions, cells synchronously proceed through S and G2 phases and become blocked at metaphase by the absence of spindle microtubules. Deletion of either *mad2* or *mad3* was additive to the *pmk1Δ* mutant, as cells leaked from mitosis with faster kinetics than the respective single deletions (Fig. 1I, J). The *slp1-mr63* allele is defective in Mad2 binding and therefore not susceptible to MCC inhibition; consequently, cells expressing this allele are highly sensitive to low doses of MBC [60]. We found that the *pmk1Δ slp1-mr63* double mutant showed an additive sensitivity to MBC relative to the single parental mutants (Fig. 1K). Together, these data suggest that the CIP MAPK Pmk1 promotes a prolonged mitotic arrest in fission yeast in response to MT damage and that its lack of function is additive to mutants in core components of the SAC (*mad2*, *mad3*, *slp1-mr63*).

### Cells lacking Pmk1 activity establish the SAC properly but display increased steady-state levels of the APC/C activator Cdc20.^Slp1^

**Fig. 2:**
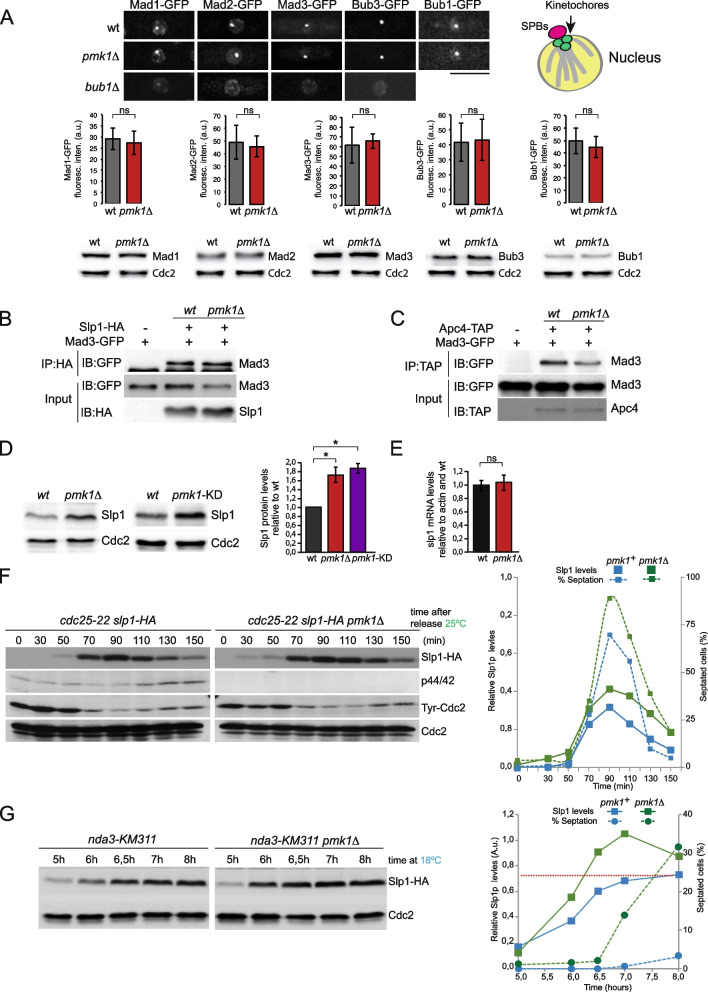
Pmk1 deletion leads to increased levels of Cdc20^Slp1^, but does not impair MCC formation or the association of MCC to APC/C.** A** Top. Microscopy images from asynchronously growing cells expressing GFP tagged version of the indicated SAC components. A kinetochore marker (Cnp1-Tomato) was used to prove proper kinetochore recruitment of SAC components (not shown). Graphs represent the average maximum signal intensity at kinetochore of the indicated markers. Error bars indicate the SD of *n*=40 cells for each condition from two independent experiments; n.s.= not statistically significant (*p* > 0.05). Bottom. Western blotting showing total protein levels of Mad1, Mad2, Mad3 Bub3, and Bub1 in the wild type and *pmk1Δ* strains. Cdc2 was used as loading control. **B** Co-IP experiment pulling down Slp1-HA and detecting Mad3-GFP. Cells were harvested from a mitotic block. Untagged Slp1 was used as a negative control, whereas Mad3- Cdc20^Slp1^ interaction in a wild type was used as a positive control. IP: Immunoprecipitation, IB: Immunoblotting. **C** Co-IP experiment pulling down APC/C component Apc4. Untagged Apc4 was used as a negative control, whereas Mad3-Apc4 interaction in a wild type was used as a positive control. **D** Western blot of total Cdc20^Slp1^ protein levels in asynchronously growing wild type, *pmk1Δ* and *pmk1-KD* cells under the same conditions. Cdc2 was used as loading control. Quantification of Cdc20^Slp1^ total levels relative to loading control is shown on the bar graph. Asterisks indicate statistical significance (*p*<0.01). **E** Cdc20^Slp1^ mRNA analysis was done by Q-PCR from asynchronously growing wild type and *pmk1Δ* cells. CT values were relativized with actin, which was used as a control, and to the wild type. Graph represents data from 3 independent repeats with 6 technical replicas; n.s. means differences are not statistically significant (*p* > 0.05). **A–E** Images show a representative example of two independent experiments.  **F** Cells from strains cdc25-22 and *pmk1Δ cdc25-2*2 were grown to O.D.= 0.3 at 25°C, shifted to 36°C for 3.5 h, and then released to permissive temperature (25°C). Samples were taken at the indicated time points. Slp1-HA was detected along with activated Pmk1 with anti-phosho-p44/42 antibody. Anti-tyrosine-Cdc2 antibody was used to follow G2/M transition. Cdc2 was used as a loading control. Percentage of septated cells and Cdc20^Slp1^ levels at each time point is indicated on the graph. **G** Western blot of mitotic Cdc20^Slp1^ levels in *nda3-KM311* and *pmk1Δ nda3-KM311* cells. Samples were taken at the indicated time points after the shift to 18°C. Cdc2 was used as loading control. Percentage of septated cells and Cdc20^Slp1^ levels at each time point are indicated on the graph. **F, G** Images show a representative example of three independent experiments

In order to elucidate the possible function of Pmk1 in mitotic regulation, we investigated whether SAC signaling was properly initiated in cells deleted for *pmk1*. As readout of initial SAC establishment and activation we quantified the fluorescence levels of endogenous GFP-tagged versions of SAC components Mad1, Mad2, Mad3, Bub1, and Bub3 at kinetochores. As shown in Fig. 2A, Bub1, Bub3, Mad1, Mad2, and Mad3 were recruited to the kinetochores in *pmk1*Δ cells with similar kinetics and signal intensities as in wild type cells. Furthermore, total protein levels of the above SAC components were similar in *pmk1*Δ and wild type cells (Fig. 2A). In contrast, the kinetochore levels of Mad1-GFP, Mad2-GFP, Mad3-GFP, and Bub3-GFP were significantly decreased in the absence of *bub1* (Fig. 2A), as previously described [61, 62].

We next checked whether the MCC is properly assembled in *pmk1*Δ cells. Chromosomally tagged Slp1-HA was immunoprecipitated from wild type or *pmk1*Δ cells, and the amount of co-purifying Mad3-GFP was used as read out of MCC formation. The levels of immunoprecipitated Mad3 were similar in wild type and *pmk1*Δ cells (Fig. 2B), suggesting that MCC is properly assembled in the absence of Pmk1. We then considered whether the defective mitotic arrest observed in *pmk1*Δ cells arises due to reduced MCC binding to the APC/C. To check this possibility, we immunoprecipitated the APC/C component Apc4 (Apc4-TAP) from control and *pmk1*Δ cells and assayed its interaction with the MCC by immunoblotting for Mad3 (Mad3-GFP). The assay was performed in the *nda3-KM311* background after 6 h of mitotic arrest, a time in which most *pmk1*Δ cells remained blocked in Metaphase. As shown in Fig. 2C, Mad3 co-purified with the APC/C, which is indicative of MCC binding. Together, these data indicate that neither kinetochore levels of SAC components, mitotic levels of MCC, nor its interaction with the APC/C are significantly impaired in *pmk1*Δ cells. However, we noticed that Mad3-GFP levels were slightly reduced in *pmk1*Δ cells, whereas Cdc20^Slp1^ levels increased (Fig. 2B, input). Indeed, we found that total Cdc20^Slp1^ protein levels in *pmk1Δ* cells or in those expressing the kinase dead allele *pmk1-KD* increased by ~ 1.7- fold with respect to wild type cells (Fig. 2D). This increase was not a consequence of transcriptional upregulation, since Cdc20^Slp1^ mRNA expression levels were similar between *pmk1*Δ cells and the wild type (Fig. 2E). Next, we analyzed the effect of Pmk1 activity on Cdc20^Slp1^ protein levels during mitotic progression. To this end, *cdc25-22 slp1-HA* and *cdc25-22 pmk1Δ slp1-HA* cells were synchronized at the G2/M transition by incubating for 4 h at 36 °C and released into mitosis by shifting the cultures to the permissive temperature (25 °C). We found that Cdc20^Slp1^ levels were upregulated during unperturbed mitosis in the absence of Pmk1 relative to control cells and this led to a slight acceleration of mitotic exit (Fig. 2F). Anti-phospho P44/42 antibody was used to detect the phosphorylated and active form of Pmk1, whereas anti-phosphotyrosine Cdc2 which detects the phosphorylated and inactive form of Cdc2 kinase was used to monitor mitotic progression. The faster kinetics of Cdc20^Slp1^ accumulation during mitosis in *pmk1*Δ cells was also observed in *nda3-KM311* strains (Fig. 2G). In these conditions, *pmk1Δ* cells have accumulated enough Cdc20^Slp1^ to activate mitotic exit 6.5 h post-arrest, as demonstrated by the increase in the septation index relative to wild-type cells (Fig. 2G, right). These observations suggest that in the absence of MAPK activity the upregulation of Cdc20^Slp1^ levels during mitosis results in a less robust SAC response.

### A minimal increase in Cdc20^Slp1^ levels results in decreased SAC robustness

To analyze whether the defective mitotic arrest of *pmk1Δ* cells is a direct consequence of the increased levels of Cdc20^Slp1^, we ectopically expressed Cdc20^Slp1^ in wild type cells from the medium-strength *nmt41* thiamine-repressible promoter (pINT-41x: *slp1-HA*, see “Methods”). Overexpression of Cdc20^Slp1^ under these conditions resulted in an increase in Cdc20^Slp1^ protein levels (Additional file 1: Figure S1A), which was very similar to the ~ 1.7-fold increase observed in *pmk1Δ* or *pmk1-KD* cells (Fig. 2D). Importantly, this resulted in growth sensitivity to MBC very similar to that elicited by *pmk1* deletion (Additional file 1: Figure S1B). Cells expressing ~ 1.7-fold Cdc20^Slp1^ levels escaped from the *nda3-KM311* mitotic arrest with similar kinetics than those of the *pmk1Δ* background (Additional file 1: Figure S1C). Moreover, either doubling Mad2 levels or decreasing Cdc20^Slp1^ levels by 40% partially suppressed the mitotic leakage caused by *pmk1* deletion in *nda3-KM311 pmk1Δ* cells (Additional file 1: Figure S1 D, E). Thus, consistent with the functional relevance of proper Cdc20^Slp1^ stoichiometry in SAC robustness [26–28], our data strongly suggest that Cdc20^Slp1^ protein upregulation is likely the cause of the defective SAC observed in the absence of CIP signaling.

### Pmk1 physically interacts with Cdc20^Slp1^ through a MAPK docking site

To gain further insight into how Cdc20^Slp1^ levels are modulated by Pmk1, we tested whether these proteins interact in vivo.

**Fig. 3:**
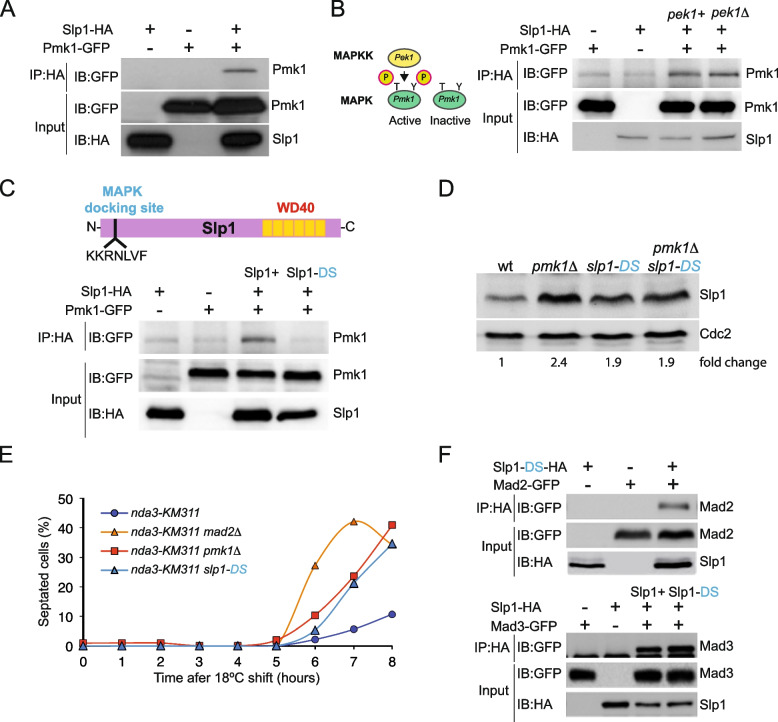
Pmk1 physically interacts with Slp1^CDC20^ through a MAPK docking site.** A** Mitotically arrested *nda3-KM311 *cells (6 h) were harvested and Pmk1- Cdc20^Slp1^ physical interaction determined by Co-IP. Untagged Cdc20^Slp1^ and Pmk1 were used as negative controls. IP: Immunoprecipitation; IB: Immunoblotting. Representative image of three independent experiments. **B **Slp1-HA was immunoprecipitated from the mitotically arrested *nda3-km2311* (*pek1+) and* *nda3-km311 pek1Δ *cells (6h) and its physical interaction with Pmk1 determined by western blotting. Representative image of two independent experiments. **C** Schematic representation of Slp1 protein indicating a putative MAPK docking site at its N-terminus. Slp1 MAPK Docking site was mutated creating the Cdc20^Slp1-DS^mutant. Pmk1 physical interaction with Cdc20^Slp1^and Cdc20^Slp1-DS^was tested by Co-IP experiment from mitotically arrested cells. **D** Cdc20^Slp1^ levels from asynchronously growing cells from the indicated strains. Numbers indicate fold change relative to wild type using Cdc2 as loading control. **E** Representative example of mitotic arrest assay of the indicated strains. Percentage of septation is shown as readout for mitotic exit/leakage; *n*=200 cells were quantitated for each strain and time point. **C–E** Representative examples of three independent experiments. **F** Cdc20^Slp1-DS^ mutant was tested by Co-IP experiment for its ability to interact with the MCC components Mad2 (above) and Mad3 (below) during a mitotic block. Untagged Cdc20^Slp1-DS^, Mad2, and Mad3 were used as negative controls. Representative example of two independent experiments

To this end, *nda3-KM311 pmk1-GFP slp1-HA *cells were pre-synchronized by HU treatment and collected after 6 h incubation at the restrictive temperature (18 °C) at which point 95% of cells are arrested in mitosis. In these conditions, Cdc20^Slp1^ co-immunoprecipitated with Pmk1 (Fig. 3A). Notably, the Pmk1-Cdc20^Slp1^ in vivo interaction was not dependent on Pmk1 activation status, since it was still detected in the absence of Pek1, the MAPKK of Pmk1 (Fig. 3B) [63].

Bioinformatic analysis revealed the presence of a putative MAPK consensus-docking site (DS) at the N-terminus of Cdc20^Slp1^ (sequence -KKRNLVF-; Fig. 3C). MAPKs DSs are characterized by a stretch of positively charged amino acids which bind their substrates and regulators with high affinity [64–66]. To test whether Pmk1- Cdc20^Slp1^ interaction requires this putative docking site, we mutated the positively charged residues from positions 19 to 25 to alanine (KKRNLVF to AAANAAF), to obtain the *slp1-DS-HA* strain (see “Methods”). As predicted, mutation of these residues abrogated Pmk1- Cdc20^Slp1^ interaction (Fig. 3C), suggesting that this motif is a bona fide MAPK docking site that mediates Pmk1-Cdc20^Slp1^ association in vivo. Moreover, cells expressing the Cdc20^*slp1−DS*^* allele* showed a modest upregulation of Cdc20^Slp1^ levels relative to wild type (1.9 fold; Fig. 3D), and, like *pmk1∆* cells, were also defective in maintaining a *nda3-KM311* mitotic arrest (Fig. 3E). Mutations introduced in the *slp1* + gene to generate the *slp1-DS* allele could affect its suitability to be sequestered into the MCC and affect SAC functionality, as occurs with the *slp1-mr63* allele [60]. Therefore, we tested the interaction between Cdc20^Slp1−DS^ and Mad2/Mad3 during a mitotic block in the *nda3-KM311* background. In these conditions, Cdc20^Slp1−DS^ interacted with the MCC components Mad2 and Mad3 during a mitotic block to a similar extent as wild type Cdc20^Slp1^ (Fig. 3F), suggesting that the defect of Cdc20^Slp1−DS^ cells in maintaining a *nda3-KM311* mitotic arrest is not due to defective MCC formation. Thus, in *S. pombe* Pmk1 interaction with Cdc20^Slp1^ N-terminal docking site fine-tunes Cdc20^Slp1^ protein levels during mitosis and consequently, proper SAC activity.

### Pmk1 activation in response to stress downregulates Cdc20^Slp1^ levels and delays mitotic exit

**Fig. 4:**
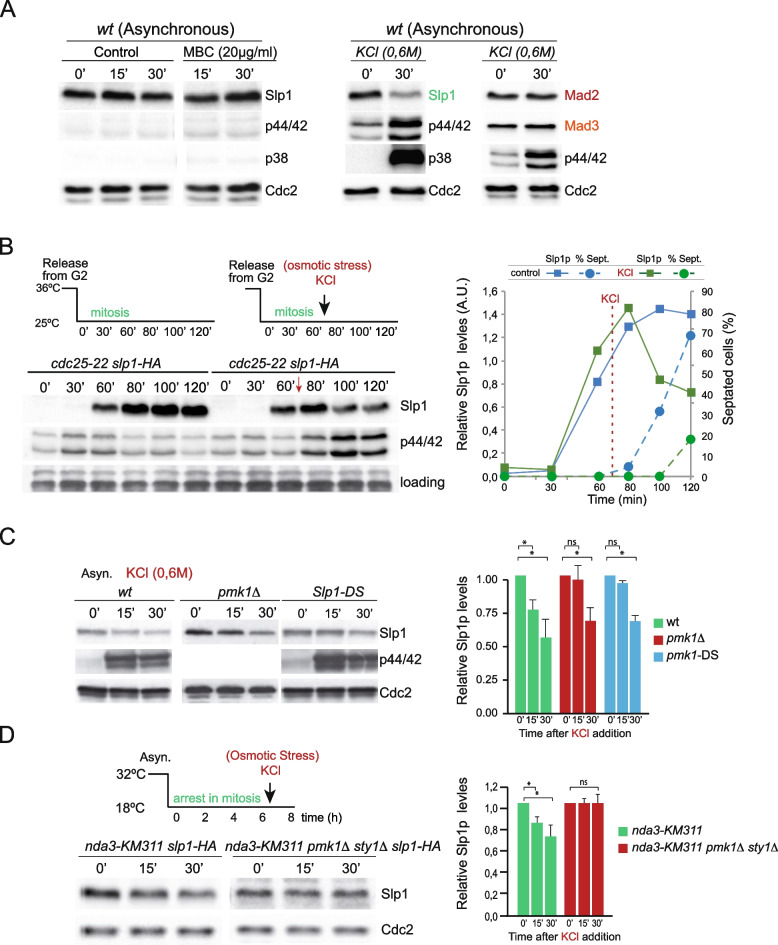
Stress elicits a rapid drop in Cdc20^Slp1^ protein levels, thus delaying mitotic exit.** A **Left, Exponentially growingwild type cells were exposed to 20µg/ml MBC and Cdc20^Slp1^ levels were determined at the indicated time points. MAPK activation was determined using anti-phospho p42/44 and anti-phospho p38 as indicative of respective Pmk1 and Sty1 activation. Right, Cdc20^Slp1^, Mad2, and Mad3 levels from asynchronously growing wild type cells were determined in control cells or after 30 min of KCl addition (0.6M). MAPK activation was determined using anti-phosho-p44/42 antibody and anti-phospho p38 as indicative of respective Pmk1 and Sty1 activation. Representative example of two independent experiments is shown.** B** Left, Cdc20^Slp1^ levels in *cdc25-22 slp1-HA* cells blocked (for 3.5 h) and then released to the permissive temperature (25°C) in unperturbed conditions (left) or after addition of KCl at time 65 min. Right, Relative Cdc20^Slp1^ levels and septation index over the course of the experiment. Representative example of three independent experiments is shown. **C** Left, Cdc20^Slp1^ levels from asynchronously growing wild type, *pmk1Δ*, and Cdc20^Slp1-DS^ cells exposed to 0.6 M KCl at the indicated times. Level of activated Pmk1 anti-phosho-p44/42 antibody along with Cdc2 as loading control is shown. Graph represents relative average levels of Cdc20^Slp1^ in each strain from two independent experiments. Asterisk denotes *p*<0.001. ns: non-significant. Representative example of two independent experiments is shown. **D **Left, Cdc20^Slp1^ levels from mitotically arrested *nda3-KM311* (6.5 h) in a wild type or *pmk1Δ sty1Δ *backgrounds, treated or untreated with 0.6M KCl. Right, Quantitation of Cdc20^Slp1^ protein levels relative to time 0 in each strain is shown. Cdc2 is used as loading control. Asterisk denotes *p*<0.001. ns: non-significant. Images are representative examples of three independent experiments

Based on the above data, we hypothesized that MBC treatment might result in Pmk1 activation and modulation of Cdc20^Slp1^ levels as a protective mechanism of mitosis control. Accordingly, we determined Cdc20^Slp1^ protein levels and Pmk1 activation status in asynchronously growing wild type cells exposed to MBC, and employing KCl as known positive control for Pmk1 activation [41]. Surprisingly, while MBC treatment did not activate Pmk1 at short incubation times, Cdc20^Slp1^ levels appeared slightly upregulated, possibly due to a partial cell synchronization at mitosis (Fig. 4A, MBC 30′). Instead, Pmk1 became progressively activated by MBC in a dose-dependent manner at longer incubation times (Additional file 2: Figure S2A). The Pmk1 activation kinetics during MBC treatment did not follow the typical sharp and transitory activation observed under saline stress, but was rather similar to Pmk1 activation observed in response to cell wall damage, which is progressive and sustained (Additional file 2: Figure S2B) [41]. As expected, KCl treatment resulted in full activation of both Pmk1 and Sty1 detected by anti-phospho p42/44 (activated Pmk1) and anti-phospho p38 (activated Sty1) antibodies, and it correlated with a marked and specific downregulation in Cdc20^Slp1^ levels, though the levels of SAC components Mad2 and Mad3 remained unaffected (Fig. 4A, right). The drop in Cdc20^Slp1^ levels during osmostress might be a consequence of a MAPK-dependent arrest in other cell cycle stages, as for instance, G2/M [67]. To test this possibility, G2 cells released into mitosis using a *cdc25-22* background were treated with osmotic stress prior to the metaphase-to-anaphase transition. Strikingly, whereas Cdc20^Slp1^ accumulated gradually during mitosis during unperturbed conditions [68], osmotic saline stress caused a sharp drop in Cdc20^Slp1^ within 15 min of addition in cells undergoing mitosis, which correlated nicely with Pmk1 activation (Fig. 4B). Consistently, stress-induced Cdc20^Slp1^ downregulation resulted in a clear delay in mitotic exit (Fig. 4B, right).

We next explored the possible dependence of Pmk1 activation and/or Pmk1- Cdc20^Slp1^ interaction through its MAPK-docking site, on Cdc20^Slp1^ downregulation during osmotic stress. Cdc20^Slp1^ levels were quickly reduced after 15 min of KCl treatment in wild type cells but remained mostly unaltered in the pmk1Δ or Cdc20^Slp1−DS^ mutants (Fig. 4C). Nevertheless, Cdc20^Slp1^ levels were reduced in both mutants 30 min after KCl addition. This delayed reduction might occur at the level of transcription or result from cell arrest at G2/M [67]. An alternative possibility is that the SAPK MAPK Sty1 might also be involved in Cdc20^Slp1^ downregulation together with Pmk1, since it also becomes strongly activated during salt stress (Fig. 4A), and its deletion phenocopies *pmk1Δ* sensitivity to MBC (Fig. 1A). Accordingly, *pmk1Δ sty1Δ* cells were unable to downregulate Cdc20^Slp1^ protein upon osmotic stress in mitotically arrested *nda3-KM311* cells (Fig. 4D). Together, these findings suggest that activation of CIP and SAPK MAPKs can contribute to rapid Cdc20^Slp1^ decay upon osmotic stress.

### Cdc20^Slp1^ downregulation under osmotic stress depends on Mad3 and the proteasome

**Fig. 5:**
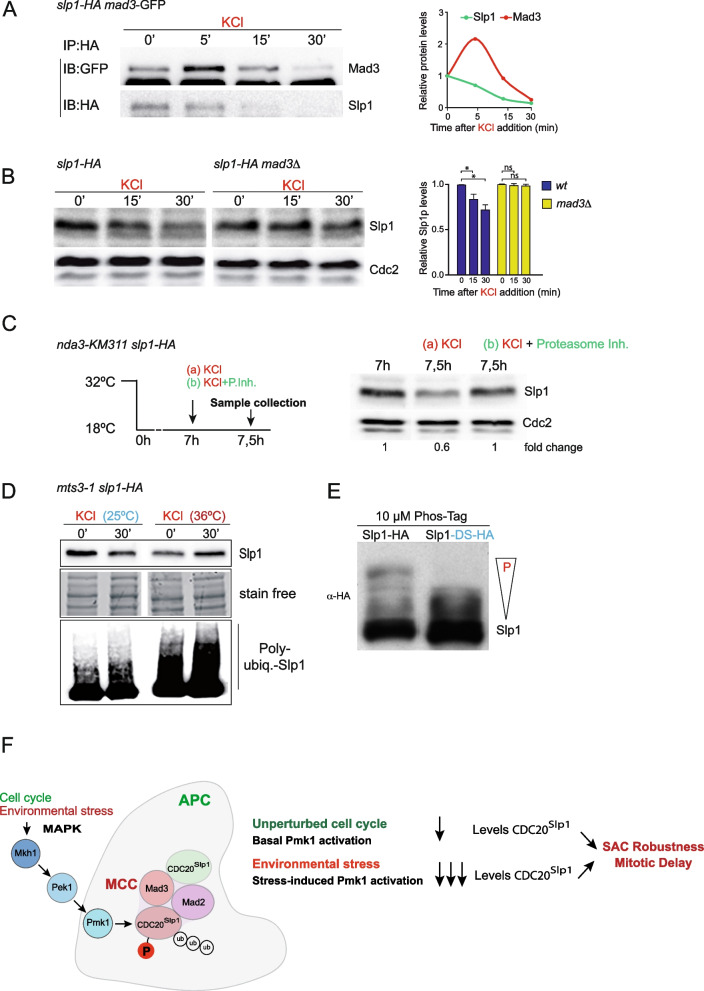
Cdc20^Slp1^ downregulation induced upon osmotic stress is prevented in the absence of Mad3 or proteasome activity. **A **Asynchronously growing cells treated with 0.6M KCl for the indicated times were harvested and Slp1-HA was immunoprecipitated and tested for its ability to bind Mad3. Time 0’ represents the Cdc20^Slp1^-Mad3 association before KCl addition. IP: Immunoprecipitation; IB: Immunoblotting. Quantification of Cdc20^Slp1^ and Mad3 levels from two independent experiments shown in left. **B **Left,Cdc20^Slp1^ protein levels were determined from asynchronously growing wild type and *mad3Δ *cells treated or untreated with 0.6M KCl at 25°C. Cdc2 was used as a loading control. Right, Average Cdc20^Slp1^ protein levels relative to time 0 from three independent experiments. Asterisk denotes *p*<0.001. ns: non-significant. **C** *nda3-KM311* cells were arrested in mitosis for 7 h and KCl, or KCl plus proteasome inhibitors (MG132 plus Bortezomib) were added to the cultures.  Levels of Cdc20^Slp1^ were analyzed at indicated time points by western blot. Cdc2 was used as loading control. Image shows a representative example of two independent experiments. Fold change in Cdc20^Slp1^ levels relative to control from the images on the left is indicated. **D** *mts3-1* cells grown at permissive and restrictive temperatures were treated with KCl 0.6M. After 30 min of KCl addition Slp1 was immunoprecipitated in each condition and the presence of poly-ubiquitinated forms of Slp1 was detected using an anti-ubiquitin antibody. **E**Cdc20^Slp1^ or Cdc20^Slp1-DS^ proteins were electrophoretically separated using 10µM of Phostag. **F** Schematic representation of MAPK-dependent Slp1 downregulation both in unperturbed cell cycles and during environmental stress. **D–E** Representative example of two independent experiments

Cdc20^Slp1^ levels are exquisitely regulated during mitosis to allow a robust SAC response [26–28, 36, 37, 61]. Cdc20^Slp1^ turnover relies on Mad3-dependent association of MCC to APC/C and is further regulated by APC/C component Apc15, which promotes Cdc20^Slp1^ poly-ubiquitination that precedes its proteasomal degradation [27, 28, 30]. To test whether the MAPK-dependent drop in Cdc20^Slp1^ levels under osmotic stress is the result of the degradation of Cdc20^Slp1^ from the pool associated with the APC/C, we performed co-IP experiments between Cdc20^Slp1^ and Mad3. Asynchronously growing cells were treated with 0.6 M of KCl and Cdc20^Slp1^-Mad3 interaction was assayed 5, 15, and 30 min after KCl addition. As shown in Fig. 5A, the Cdc20Slp1-Mad3 association increased just 5 min after osmotic stress and was followed by a fast Cdc20Slp1 decay which correlated with a decrease in Mad3- Cdc20^Slp1^ interaction (Fig. 5A). Mad3 is required for the association of MCC to APC/C [27, 28, 69], and this interaction might be required to maintain proper Cdc20^Slp1^ levels not only during unperturbed mitosis, but also in response to stress. To analyze this possibility, we determined Cdc20^Slp1^ decay under osmotic stress in the absence of *mad3*. In contrast to wild-type cells, Cdc20^Slp1^ downregulation during stress was totally abolished in the absence of Mad3 (Fig. 5B). We also explored whether Cdc20^Slp1^ was specifically degraded during mitosis by the proteasome during osmotic stress by employing *nda3-KM311 slp1-HA* cells pre-synchronized with HU at 30 °C, and then shifted to the restrictive temperature (18 °C). Mitotic-arrested cells were exposed to osmotic stress (KCl) in the presence or absence of a proteasome inhibitor cocktail (Bortezomib plus MG132), and Cdc20^Slp1^ was detected by western blot. As shown in Fig. 5C, proteasome inhibition prevented Cdc20^Slp1^ downregulation in response to osmotic stress. This effect was also observed when proteasome function was impaired by using a mutant in the proteasome essential component Rpn12 (*mts3-1* mutant, Fig. 5D), and resulted in enhanced presence of ubiquitinated forms of immunoprecipitated Cdc20^Slp1^ during osmotic stress at the restrictive temperature (Fig. 5D).

Multiple attempts to detect direct in vitro phosphorylation of Cdc20^Slp1^ by Pmk1 were unsuccessful (data not shown). However, analysis by Phos-tag SDS-PAGE of immunoprecipitates from cells arrested in mitosis by the *nda3-KM311* allele showed significant changes in the electrophoretic mobility of the Cdc20^Slp1−DS^ isoform relative to Cdc20^Slp1^ (Fig. 5E). This suggests that the Cdc20^Slp1^ docking site is required for its phosphorylation in vivo and is consistent with a possible involvement of Pmk1 and/or Sty1 in this posttranslational modification, which might affect its in vivo turnover and/or half-life (Fig. 5G).

## Discussion

### Deletion of Pmk1 leads to reduced SAC robustness

Consistent with its role as activator of the metaphase to anaphase transition, Cdc20^Slp1^ protein peaks during mitosis and is rapidly degraded by the proteasome in an APC/C-Cdh1-dependent manner after mitotic exit to reset its levels in preparation for the next cell cycle. Importantly, maintenance of the mitotic checkpoint, which becomes critical for cell survival in conditions of defective chromosome capture, also requires targeted Cdc20^Slp1^ ubiquitination by APC/C-Cdc20^Slp1^, followed by its proteasomal degradation. Cdc20^Slp1^ proteostasis is therefore essential to fine-tune this checkpoint and prevent mitotic slippage and the generation of aneuploidies [27, 28, 30, 69, 70]. Consistently, increasing Cdc20^Slp1^ protein levels both in yeast and in higher eukaryotes leads to a less robust mitotic checkpoint, as the MCC is not able to counteract excess of Cdc20 molecules [31, 32, 70, 71]. Importantly, CDC20 upregulation is associated with several types of cancer including breast and colon cancer and has been involved in aggressive tumor progression and poor prognosis in gastric cancer and primary non-small cell lung cancer [72]. Conversely, reducing Cdc20^Slp1^ levels increases SAC robustness, as Cdc20^Slp1^ would be limiting to activate anaphase [71]. Our work demonstrates that deletion of MAPK Pmk1 leads to upregulated Cdc20^Slp1^ protein levels which leads to accelerated mitotic exit (Fig. 2F-G). In addition, *pmk1Δ* cells showed a twofold increased rate of minichromosome loss during unperturbed mitosis (Fig. 1F). Lack of Pmk1 function is phenotypically cryptic with regard to maintenance of Cdc20^Slp1^ levels under normal growth conditions, as chromosome capture by the mitotic spindle occurs efficiently. However, its role becomes essential when chromosome capture is compromised by a dysfunctional mitotic spindle, as demonstrated by *pmk1Δ* hypersensitivity to MBC, and the inability of *pmk1Δ* cells to maintain a metaphase arrest resulting in a premature slippage relative to wild-type cells in the *nda3-Km311* mutant background (Fig. 2G). Therefore, we propose a novel function for this MAPK in the maintenance of mitotic checkpoint which involves Cdc20^Slp1^ proteostasis (Fig. 5F).

### MAPK regulation of Cdc20^Slp1^ levels

The regulation of mitotic progression by Pmk1 requires a MAPK docking site present in the N-Terminus of Cdc20^Slp1^, since mutation of this domain leads to upregulated Cdc20^Slp1^ protein levels and decreased SAC robustness, phenocopying *pmk1* deletion. This mechanism might involve Cdc20^Slp1^ phosphorylation by Pmk1 as suggested by recent data [73], and the evident differences in migration of Cdc20^Slp1^ isoforms by Phos-tag analysis between wild type and Cdc20^Slp1−DS^. In *Xenopus laevis* egg extracts, CDC20 is phosphorylated at Ser 50, Thr 64, Thr 68, and Thr 79 during mitosis, and two of these phosphorylation sites (Thr 64 and Thr 68) have been proven to be MAPK-dependent. A CDC20 mutant in which any of the four phosphorylation sites were replaced by non-phosphorylatable amino acids fails to respond to the spindle checkpoint signal due to decreased affinity of CDC20 for Mad2 and Bub1R [45]. Importantly, CDC20 degradation mediated by MAPK p38 has been proposed as a mechanism to maintain a metaphase arrest of human cells treated with the carcinogen Cadmium [74]. Thus, it is possible that phosphorylation and regulation of CDC20 levels by MAPKs is an evolutionarily conserved mechanism to adjust mitotic exit to extracellular or environmental stimuli. In addition, we detected increased accumulation of Cdc20^Slp1^ in *pmk1Δ cdc25-22* cells released into mitosis, and a higher rate of minichromosome loss compared to wild type cells, suggesting that this mechanism of regulation also operates during unperturbed cell growth.

Phosphorylation of CDC20 by Cdk1/cyclin B complexes hampers its ability to bind and activate APC/C in mitotic mammalian cells and Xenopus egg extracts [36–38, 45, 75, 76] raising the possibility that phosphorylation of Cdc20^Slp1^ by Pmk1 may similarly decrease its affinity for APC/C. However, we found that Pmk1 instead regulates Cdc20^Slp1^proteasomal degradation at the MCC-APC/C. *pmk1Δ* cells show increased accumulation of Cdc20^Slp1^ during unperturbed mitosis and during SAC activation (Fig. 2). Activation of Pmk1 by osmotic stress leads to a rapid decay in Cdc20^Slp1^ protein levels (Fig. 4) which is dependent on Mad3 (Fig. 5B) suggesting that proteolysis of Cdc20^Slp1^ depends on MCC and/or MCC binding to APC/C. Consistently, the decay in Cdc20^Slp1^ levels upon osmotic stress is abolished in the presence of proteasome inhibitors, and ubiquitinated forms of Cdc20^Slp1^ accumulated in these conditions if proteolysis is blocked by using a thermosensitive proteasome mutant (Fig. 5C, D).

In fission yeast, it has been shown that proper Cdc20^Slp1^ turnover requires the association of MCC to APC/C, and the APC/C subunit Apc15 that promotes Cdc20^Slp1^ ubiquitination and degradation by the proteasome [27, 69]. Since MCC associates with the APC/C independently of Pmk1 (Fig. 2), the function of Pmk1 is likely related to the last stage of Cdc20^Slp1^ ubiquitination and degradation). Whether Pmk1 directly phosphorylates Cdc20^Slp1^ and the molecular mechanism by which this phosphorylation promotes Cdc20^Slp1^ ubiquitination and/or degradation will require further investigation.

### Cdc20^Slp1^ downregulation upon stress-induced MAPK activation

Treatment of fission yeast cells with KCl, which provokes a strong osmotic stress response and quick Pmk1 activation, resulted in a rapid decrease in Cdc20^Slp1^ protein levels. The drop of Cdc20^Slp1^ during KCl treatment, which was partially suppressed by *pmk1* deletion or in a Cdc20^Slp1−DS^ docking-site mutant, was fully abolished in the absence of both Pmk1 and Sty1 MAPKs. This suggests that Sty1 exerts further levels of regulation of the mitotic checkpoint in addition to Pmk1. Importantly, Cdc20^Slp1^ downregulation upon osmotic stress leads to an extension of the mitotic phase and delays cytokinesis. Thus, the phenotypic suppression of MBC sensitivity by osmotic stress initially described in our screen (Fig. 1), likely results from Cdc20^Slp1^ downregulation induced by MAPKs (Fig. 5F).

### Biological significance of delaying mitotic exit under osmotic stress

Extending the timing of cell cycle transitions through checkpoint mechanisms occurs normally as a function of a particular uncompleted process like, for instance, ongoing DNA repair, in the case of the G2/M DNA damage checkpoint, or unattached or tension-less kinetochores, in the mitotic checkpoint. It is known that osmotic stress freezes interphase microtubules [77], and thus it might also alter spindle microtubule dynamics compromising chromosome capture and inducing a conventional SAC-dependent delay of the metaphase to anaphase transition. Alternatively, the drastic drop in Cdc20^Slp1^ protein levels upon osmotic stress could be a cellular protective response rather than a response to an unfulfilled process. Osmotic stress elicits intracellular water imbalance that directly impacts overall protein concentration within the cell [78, 79]. Intriguingly, as the SAC operates through exquisitely regulated stoichiometric relationships between many of their signaling components, minimal changes in their relative protein concentrations during water imbalance could alter SAC signaling function. In this context, Pmk1-dependent extension of mitosis induced by osmotic stress might allow cells to restore their water equilibrium before commitment to mitotic exit and cytokinesis.

Pmk1 has also been recently proposed to act as a negative regulator of cytokinesis by a mechanism that involves the Septum Initiation Network (SIN), a signaling cascade that temporally coordinates exit from mitosis and the activation of cytokinesis [80]. Consistently, Pmk1 deletion shows severe defects during cytokinesis, especially under osmotic stress, and does not respond to threats in the cytokinetic machinery, similarly to data shown in this work with respect to the mitotic spindle). Therefore, it is likely that Pmk1 contributes to signal environmental stimuli to both the mitotic and cytokinesis surveillance mechanisms to maintain cellular homeostasis under adverse osmotic conditions.

### Overall strategies to survive in stressful conditions

Adaptation to environmental stress operates through multiple cellular mechanisms to provide cells with a positive and selective growth advantage [81]. These adaptation mechanisms involve transient changes in gene expression, as well as changes in protein stability, localization, and activity, or more invasive mechanisms like genome instability caused by mutations, chromosome rearrangements, or by the generation of aneuploidies. It has been recently shown that hypomorphic mutations or deletion in MAPK signaling genes are enriched during the non-proliferative state of quiescence [82]. Mutation in these kinases leads to chromosome instability, and this might be a main mechanism to generate genetic diversity in adverse conditions such as during chronological aging. However, rapid and transient MAPK activation in response to adverse conditions seems to be the preferred surveillance mechanism to adjust cell cycle transitions and maintain homeostasis during cellular proliferation.

Given the oncogenic role for CDC20-APC/C in tumor progression as well as its role in drug resistance, the inhibition of CDC20-APC/C activity or the induced degradation of CDC20 are emerging as alternative therapeutic strategies to control cancer [83, 84]. Thus, the discovery of new layers of CDC20 regulation might help to develop new strategies to target this key cell cycle regulator in cancer therapy.

## Conclusions

This work reveals a novel function of MAPK in the control of SAC robustness through regulation of Cdc20^slp1^ levels. This control, which operates during every cell cycle, allows cells a rapid response to environmental stress during mitosis and contributes to maintain genomic stability.

## Methods

### Schizosaccharomyces pombe growth media

Culture media used in this work for the growth and maintenance of *S. pombe* are described elsewhere [85]. Rich medium yeast extract (YES) was supplemented with adenine, leucine, histidine, and uridine to a final concentration of 300 mg/L. Antibiotics geneticin (G-418, Life Technologies #K4000), nourseothricin (clon Nat, Werner Bioagents, #5001000), or Hygromycin B (Roche, #10843555001) were added to the media at a final concentration of 100 μg/ml when required. Edinburgh Minimal Medium (EMM) containing the necessary supplements was used to select *S. pombe* strains with auxotrophic markers. Sporulation medium (SPA) plates were used to induce conjugation and sporulation. Strains used in this study are listed in Additional file 3.Table S1**.**

### Cell culture conditions

All the experiments described were performed with exponential phase cultures with an optical density between 0.2 and 0.8 measured at 595 nm. Ordinarily *S. pombe* liquid cultures were grown at 30 °C in orbital shakers. For the *cdc25-22* thermosensitive mutants, permissive temperature was 25 °C and restrictive temperature was 36 °C. For the *nda3-KM311* cold-sensitive mutant, permissive temperature was 32 °C and restrictive temperature was 18 °C.

### Mitotic arrests

For the metaphase block using the cold-sensitive tubulin mutant, *nda3-KM311* cells were grown overnight in YES media at 32 °C to early-log phase and 12 mM of hydroxyurea (HU) was added to the cultures. After 3.5 h of incubation at 32 °C, HU was washed out and cells were shifted to pre-cool YES at 18 °C and incubated at that temperature until sample collection. For the immunoprecipitations and co-immunoprecipitations experiment incubation time at 18 °C was 6 h unless otherwise indicated. Cells were fixed in 70% ice-cold ethanol at each indicated time point. To determine the septation index, cells were fixed in 70% ice-cold ethanol at each indicated time point and mounted with a DAPI-calcofluor staining solution. In order to synchronize cells in mitosis, thermo-sensitive *cdc25-22* cells were grown overnight in YES medium at 25 °C to mid-log phase and then shifted to 36 °C for 3.5 h to arrest them in G2. In order to synchronously release cells into mitosis, cultures were rapidly cooled down to 25 °C in iced-water and then incubated at 25 °C for the experimental time course. Progression through mitosis was monitored by DAPI and calcofluor staining scoring septated cells.

### Spot assays

All strains were grown to mid-log phase (identical OD) and 4 serial dilutions (1/10, 1/100, 1/1000, and 1/10,000) were spotted into YES plates and YES plates containing the indicated compounds. Unless otherwise stated, plates were incubated at 25 °C during 3–5 days before imaging.

### Minichromosome loss assay

This assay was performed using wild type and *pmk1Δ* cells as described in Niwa, 2018 [86]. Briefly, wild type and *pmk1Δ* cells expressing minichromosome 16 were grown at 25 °C in EMMG media (low adenine media: 5 mg/ml adenine sulfate) to mid-log phase. Cells were diluted to 1 × 10^4^cells/mL with EMMG and plated in serial dilutions in EMMG-agar plates. The frequency of half-sectored colonies was determined after incubation of the cells at 25 °C for 3–4 days.

### Microscopy

For in vivo imaging, cell cultures were grown to exponential phase. Cells were pelleted by centrifugation at 3000 rpm and resuspended in the residual media. Cells were placed on microslide wells (Ibidi, # 80,821) coated with soybean lectin (Sigma, #L1395). Images were acquired using a Spinning-disk confocal microscope (IX-81; Olympus; CoolSnap HQ2 camera, Plan Apochromat 100x-1.4 NA objective [Roper Scientific]). MetaMorph software was used for image acquisition. Temperature was stably controlled at 25 °C during imaging. For fixed cells, a DAPI-calcofluor staining was used. Cells fixed with 70% ethanol were washed with PBS and pellet was resuspended in 5 μl of the DAPI/calcofluor mounting solution (100 μl Mounting solution contains: 38 μl glycerol 50%, 46.5 μl H_2_O, 10 μl Antifade (p-phenylenediamine, Sigma #P6001; 10 mg/ml in phosphate-buffered saline pH 8.2), 3 μl DAPI (4, 6-diamidino-2-phenylindole, Sigma #D1388; 0.1 mg/ml), and 2.5 μl calcofluor white (Sigma #F6259; 0.35 mg/ml in H_2_O).

### Preparation of cell extracts

Routinely, cells were pelleted by centrifugation, washed once with cold PBS (Phosphate Buffered Saline: 10 mM Na2HPO4, 2 mM KH2HPO4, 137 mM NaCl y 2,7 mM KCl) and stored at − 80 °C until processed. Cell homogenates were prepared under native conditions employing chilled acid-washed glass beads (Sigma, # G8772), lysis buffer (10% glycerol, 50 mM Tris–HCl, pH 7.5, 150 mM NaCl, 0.1% Nonidet P-40) plus specific protease and phosphatase inhibitor (Complete EDTA-free Protease Inhibitor Cocktail; Sigma, #11873580001; 150 mM NaF, Sigma #S7920, and 150 mM Phenylmethylsulphonyl fluoride, Sigma, #52332). Cells were resuspended in lysis buffer and bead-beaten at 4 °C four times during 30 s in the FastPrep-24 (MP Biomedicals). The lysates were cleared by centrifugation at 13,000 rpm for 10 min at 4 °C.

### Protein electrophoresis and Western blot

Protein electrophoresis was performed using SDS–polyacrylamide gels (SDS-PAGE) and Phos-Tag gels (Wako Laboratory Chemicals, # 300–93523). Protein samples were prepared by adding SDS-Sample Buffer 2X (4% SDS, 100 mM Tris–HCl pH 6, 8, 20% glycerol, 0.2% Bromophenol Blue, 200 mM DTT, dithiothreitol) and heating at 95 °C for 5 min before loading onto regular 10% acrylamide gels or 6% acrylamide in the case of Phos-Tag gels. Proteins were transferred to nitrocellulose membranes and blocked with PBS + Tween-20 + 5% (w/v) dry milk for 30 min or 10 mM Tris–HCl, 150 mM NaCl, pH 7.5 (TBS) + 0.05% Tween-20 + 5% (w/v) dry milk in the case of PhosTag gels. Blots were incubated o/n with the indicated primary antibody followed by incubation with anti-IgG-HRP (HRP, horseradish peroxidase) secondary antibody for 1 h at room temperature. For protein detection, Super Signal West Femto (ThermoScientific, #34,095) or Clarity Western ECL (Bio-Rad, #1,705,060) and the Chemidoc Imaging system (Bio-Rad) were used.

### Immunoprecipitation

Protein extracts (6–10 mg) were incubated with 10 μl of Anti-HA 12CA5 antibody (Roche, #11583816001) for 1 h at 4 °C, then 25 μl of Protein a Agarose was added (Roche, #11134515001). Samples were incubated overnight with orbital rotation at 4 °C. Beads were washed six times with co-IP Washing Buffer (50 mM Tris–HCl, pH 7.5; 500 mM sodium chloride; 0.1% Nonidet P-40). Bound proteins were solubilized by the addition of SDS-sample buffer and heating at 95 °C for 8 min. Alternatively, HA-tagged proteins were immunoprecipitated using anti-HA Magnetic beads (Pierce, #88836). After 2 h incubation at 4 °C, beads were washed six times using a magnetic rack with co-IP Washing Buffer. Bound proteins were solubilized with 25 μl of HA Synthetic Peptide (Sigma, #11666975001) at 37 °C for 30 min. For pulling down GFP-tagged proteins, 6–10 mg total protein were incubated with 10 μl with Anti-GFP (Roche, 11814460001) for 1 h at 4 °C and, subsequently, 25 μl of Dynabeads Goat Anti-Mouse IgG (Invitrogen, #11,033) was added. Samples were incubated overnight with orbital rotation at 4 °C. Magnetic beads were washed six times using a magnetic rack with co-IP Washing Buffer. Bound proteins were solubilized by the addition of SDS-sample buffer heated at 95 °C for 8 min.

### Proteasome inhibition assay

*nda3-Km311* cells were incubated at the restrictive temperature (18 °C), and proteasome inhibitors Bortezomib (Selleckchem, #S1013) and MG132 (MedChem Express, #HY-13259) were added directly to the culture to a final concentration of 250 μM and 50 μM respectively. PR-619, a reversible inhibitor of deubiquitinylating enzymes (Selleckchem, #S7130), was also added to a final concentration of 5 μM. Additionally, lysis buffer used for extract preparation was supplemented with 50 μM MG132, 50 μM PR-619, and 20mN of the inhibitor of cysteine peptidases NEM (Selleckchem, #S3692).

Alternatively, *mts3-1* cells expressing Slp1-HA were grown in YES to mid-log phase at permissive (25 °C) temperature and then shifted to restrictive (36 °C) temperature for 3 h. 0.6 M KCl was added to the cultures 30 min before harvesting. Cells were harvested by centrifugation, and native extracts were prepared and used to immunoprecipitate Slp1-HA (as above). Samples were immunoblotted with Anti-HA 12CA5 antibody to detect Slp1-HA and with anti-Ubiquitin antibody (Enzo, BML-PW8810) to detect ubiquitinated forms of Slp1-HA.

### Total RNA isolation, cDNA synthesis, and quantitative PCR

Total RNA was isolated using the RNeasy Mini Kit (Quiagen, #74104) following the manufacturer’s instructions followed by DNase treatment (Quiagen, #79254). cDNA synthesis and the quantitative PCR were done simultaneously using the iTaq Universal SYBR Green One-Step RT-qPCR Kit (Bio-Rad, #172–5150). Reaction mix preparation and Thermal Cycling Protocol were performed following manufacturer’s instructions. cDNA was quantitatively measured in triplicate with the ABI Prism 7000 sequence detection system. Actin was used as the normalizing gene. To perform the analysis of the data, ΔCt value for each sample and primer is obtained as follows: Ct for a primer minus Ct for the normalizing control (actin) under a particular experimental condition (asynchronous cultures). Then, the values are referred to the control condition, obtaining the ΔΔCt value (sample ΔCt − control ΔCt = ΔΔCt). Finally, quantification of RNA levels was performed using the relative quantification (RQ), calculated as follows: RQ = 2 − ΔΔCt. We consider a RQ significant when there is a minimum of twofold change: RQ of more than 2 or less than 0.5.

### Statistical analysis

Graphs and statistical analyses were performed with Prism 5.0 (GraphPad Software) and Microsoft Excel. Unless otherwise stated, graphs represent mean and error bars represent SD. “*n*” is the total number of cells scored from two or three independent experiments. Statistical comparison between two groups was performed by unpaired Student’s *t* test, considering non-significant (n.s.), two-tailed *P*-values exceeding 0.05.

### Supplementary Information

**Additional file 1: Figure S1.** A minimal increase in Cdc20^Slp1^ levels causes sensitivity to MBC and defective mitotic arrest. A. Slp1 protein level was determined from asynchronously growing cells of the indicated genotypes. Cdc2 was used as a loading control. B. Spot growth assay of the indicated strains and condition. C-E. Representative examples of mitotic arrest assay of the indicated strains. Cell cultures were grown at permissive temperature (32ºC) and then shifted to restrictive temperature (18ºC). Samples were taken every hour and the septation index calculated form more than 200 cells in each time point and condition. Representative examples of two independent experiments are shown.

**Additional file 2: Figure S2.** Activation kinetics of MAPKs Pmk1 in response to microtubule damage. A. Left, Cells expressing *pmk1-HA* were treated with increasing concentrations of MBC (5, 10, 20µg/ml) and levels of Pmk1 activation were determined by anti-phosho-p44/42 antibody at the indicated time points. Total Pmk1 levels were determined by using an anti HA antibody. Right, graph showing levels of Pmk1 activation from three independent experiments. B. Cells expressing *pmk1-HA* were exposed to DMSO or 25µg/ml of MBC and the kinetics of Pmk1 activation was determined by using anti-phosho-p44/42 antibody. Total Pmk1 levels were determined at the indicated times by using an anti HA antibody. Image shows a representative example of three independent experiments.

**Additional file 3.** **Table S1.** List of strains used in this study.

**Additional file 4.** Original blots used in this study.

## Data Availability

All data generated or analyzed during this study are included in this published article and its supplementary information files. The strains, reagents, and datasets used and/or analyzed during the current study are available from the corresponding author on reasonable request.

## Abbreviations

MAPK: Mitogen-activated protein kinase
CIP: Cell integrity pathway
SAPK: Stress-activated protein kinase pathway
SAC: Spindle assembly checkpoint
APC/C: Anaphase-promoting complex/cyclosome
O-Mad2: Open Mad2
C-Mad2: Closed Mad2
MT: Microtubule
MBC: Carbendazim
HU: Hydroxyurea
MCC: Mitotic checkpoint complex
