# Supplementary figures and images for: MAPK-dependent control of mitotic progression in S. pombe
Source: BMC Biol. 2024 Mar 25;22:71. doi: 10.1186/s12915-024-01865-6 (PMC10962199; doi:10.1186/s12915-024-01865-6)

A

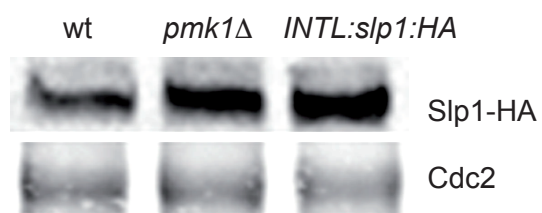

B

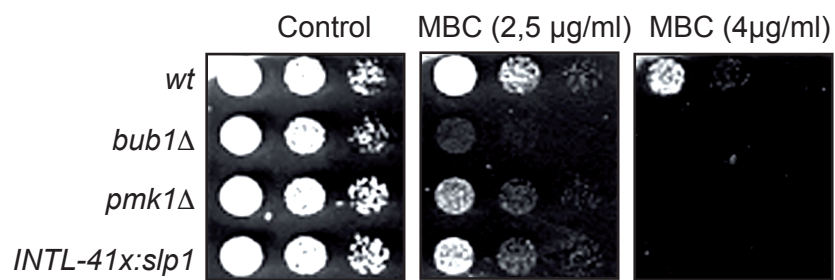

C

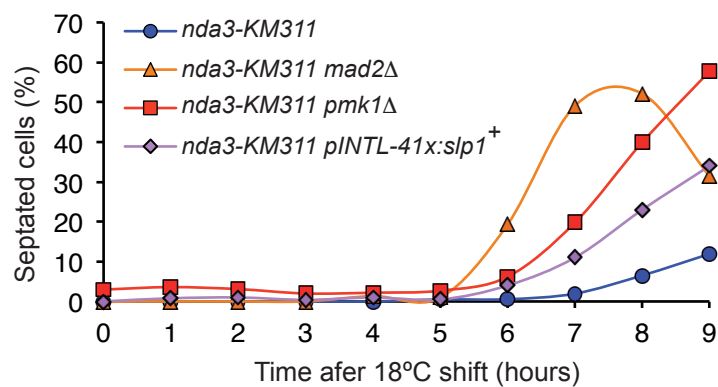

D

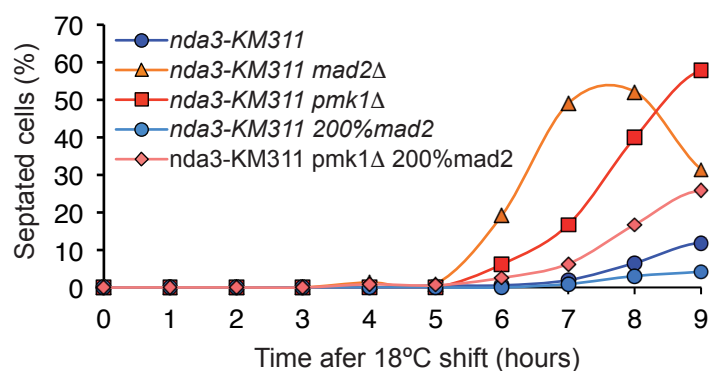

E

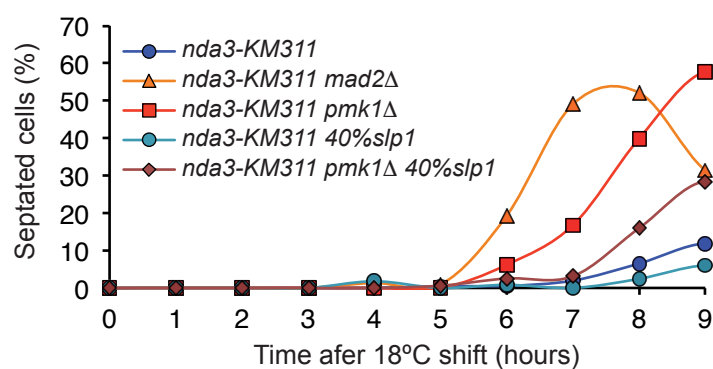

Iglesias et al Figure S1

Supplement: Supplementary file 1 — Additional file 1: Figure S1. A minimal increase in Cdc20Slp1 levels causes sensitivity to MBC and defective mitotic arrest. A. Slp1 protein level was determined from asynchronously growing cells of the indicated genotypes. Cdc2 was used as a loading control. B. Spot growth assay of the indicated strains and condition. C-E. Representative examples of mitotic arrest assay of the indicated strains. Cell cultures were grown at permissive temperature (32ºC) and then shifted to restrictive temperature (18ºC). Samples were taken every hour and the septation index calculated form more than 200 cells in each time point and condition. Representative examples of two independent experiments are shown. [file 12915_2024_1865_MOESM1_ESM.pdf]

A

*pmk1-HA-6His*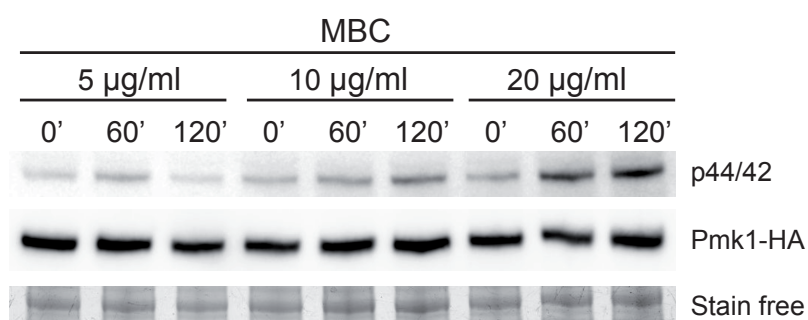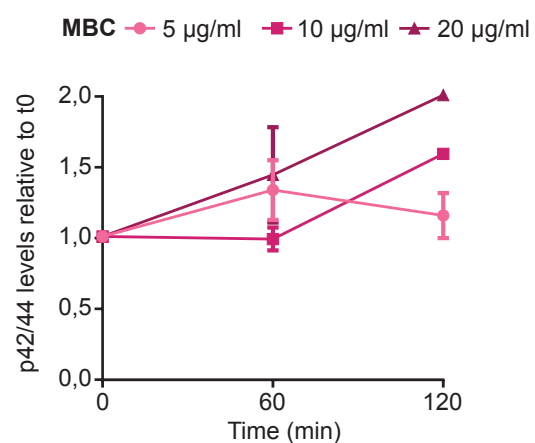

B

*pmk1-HA-6His*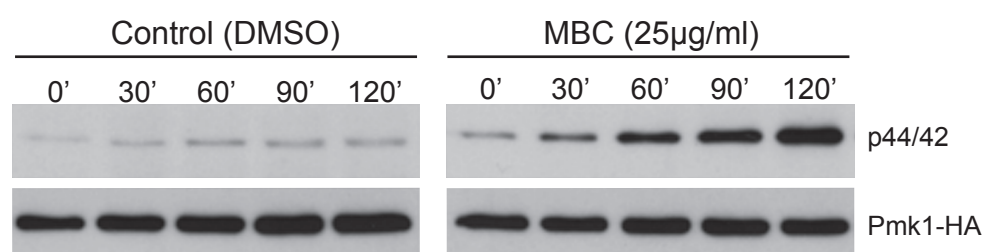

Supplement: Supplementary file 2 — Additional file 2: Figure S2. Activation kinetics of MAPKs Pmk1 in response to microtubule damage. A. Left, Cells expressing pmk1-HA were treated with increasing concentrations of MBC (5, 10, 20µg/ml) and levels of Pmk1 activation were determined by anti-phosho-p44/42 antibody at the indicated time points. Total Pmk1 levels were determined by using an anti HA antibody. Right, graph showing levels of Pmk1 activation from three independent experiments. B. Cells expressing pmk1-HA were exposed to DMSO or 25µg/ml of MBC and the kinetics of Pmk1 activation was determined by using anti-phosho-p44/42 antibody. Total Pmk1 levels were determined at the indicated times by using an anti HA antibody. Image shows a representative example of three independent experiments. [file 12915_2024_1865_MOESM2_ESM.pdf]
