# Supplementary material for: MAPK-dependent control of mitotic progression in S. pombe
Source: BMC Biol. 2024 Mar 25;22:71. doi: 10.1186/s12915-024-01865-6 (PMC10962199; doi:10.1186/s12915-024-01865-6)
Supplement: Supplementary file 3 — Additional file 3. Table S1. List of strains used in this study. [file 12915_2024_1865_MOESM3_ESM.pdf]

TABLE S1. *S. pombe* strains used in this study

Strain	Genotype	Source
RD1936	*h+ ura4-D18 leu1-32*	P. Nurse's Lab
RD 2806	*h- bub1::leu1+ ura4-D18 leu1-32*	I. Hagan's Lab
RD 1000	*h+ pmk1:: ura4+ ura4-D18 leu1-32*	M. Balasubramanian's Lab
RD 1215	*h+ mkh1::ura4+ leu1-32 ura4-D18*	M. Balasubramanian's Lab
RD 3611	*h- pek1::ura4+ leu1-32 ura4-D18*	M. Balasubramanian's Lab
RD 1057	*h- pek1DD:ura4+ nda3-KM311 ade6-M216*	J. Cansado's Lab
RD 3321	*h- pmk1::KanMX pmk1HA6His (K52E):leu1+ leu1-32 ura4-D18 ade6-M216*	J. Cansado's Lab
RD 363	*h- mad2::ura4+ ura4-D18 leu1-32*	P. Nurse's Lab
RD 988	*h mad3::ura4+ ura4-D18*	T. Toda's Lab
RD 989	*h mad1:: ura4+ ura4-D18 leu1-32*	P. Nurse's Lab
RD 981	*h mph1:: ura4+ ura4-D18 leu1-32*	P. Nurse's Lab
RD 3407	*h+ pmk1::KanMX mad2::ura4+*	This work
RD 3330	*h+ pmk1::ura4+ mad3::ura4+ leu1-32 ura4-D18*	This work
RD 488	*h- nda3-KM311 ura4-D18 leu1-32*	P. Nurse's Lab
RD 3214	*h+ mad2:: ura4+ nda3-KM311 ura4-D18*	This work
RD 5727	*h+ pmk1::ura4+ nda3-KM311*	This work
RD 3989	*h pmk1::ura4+ mad2::ura4+ nda3-KM311*	This work
RD 6085	*h pmk1::ura4+ mad3::ura4+ nda3-KM311*	This work
RD 4561	*h- slp1-mr63 leu1-32 ura4-D18*	K. Hardwick's Lab
RD 4559	*h- pmk1::ura4+ slp1-mr63 leu1-32ura4-D18*	This study
RD 2261	*h+ mad1-GFP:his+ cnp1-mCherry:KanMX leu1-32 ura4-D18*	R.R. Daga's Lab
RD 3132	*h+ pmk1::ura4+mad1-GFP:his+ cnp1-mCherry:KanMX ura4-D18*	This work
RD 368	*h- mad2-GFP:KanMX ura4-D18 leu1-32*	P. Nurse's Lab
RD 3317	*h- pmk1::ura4+ mad2-GFP:KanMX ura4-D18*	This work
RD 2121	*h+ mad3-GFP:his3+ leu1-32 ura4-D18 ade6-M216*	K. Hardwick's Lab
RD 4067	*h pmk1::ura4+ mad3-GFP:his leu1-32*	This study
RD 408	*h- bub1-GFP:KanMX leu1-32 ura4-D18*	P. Nurse's Lab
RD1005	*h- pmk1::ura4+ bub1-GFP:KanMX leu1-32 ura4-D18*	R.R. Daga's Lab
RD 2352	*h bub3-GFP:his+ leu1-32 ura4-D18*	K. Hardwick's Lab
RD 4281	*h pmk1::ura4+ bub3-GFP:His ura4-D18*	This work
RD 3173	*h- bub1::ura4+ mad1-GFP:his+ cnp1-mCherry:KanMX ura4-D18*	This work
RD 2213	*h- bub1::leu1+ mad2-GFP: KanMX cnp1-mCherry:KanMX ura4-D18 leu1-32*	R.R. Daga's Lab
RD 4069	*h bub1::ura4+ mad3-GFP:his+ ura4-D18 leu1-32*	This work
RD 6802	*h bub1::ura4+ Bub3-GFP:his+ cnp1-mCherry:KanMX ura4-D18*	This work
RD 5827	*h slp1-HA: KanMX mad3-GFP:his+ nda3-KM311 ura4-D18 leu1-32*	This work
RD 5828	*h pmk1::ura4+ slp1-HA: KanMX mad3-GFP:his+ nda3-KM311 ura4-D18 leu1-32*	This work

RD 5109	*h lid1-TAP:KanMX mad3-GFP:his+ nda3-KM311 ura4-D18 leu1-32*	This work
RD 5111	*h pmk1::ura4+ lid1-TAP:KanMX mad3-GFP:his+ nda3-KM311 ura4-D18 leu1-32*	This work
RD 2315	*h+ slp1-HA:KanMX leu1-32 ura4-D18*	K. Hardwick's Lab
RD 3459	*h+ pmk1:: ura4+ slp1-HA:KanMX leu1-32*	This work
RD 5729	*h cdc25-22 slp1-HA:KanMX ade6*	This work
RD 3675	*h- pmk1:: ura4+ cdc25-22 slp1-HA:KanMX*	This work
RD 5098	*h+ slp1-HA:KanMX nda3-KM311*	This work
RD 5099	*h+ pmk1:: ura4+ slp1-HA:KanMX nda3-KM311*	This work
RD 4077	*h- pINTL41x-slp1-HA:ura4+*	This work
RD 4117	*h- pINTL41x-slp1-HA:ura4+ nda3-KM311 leu1-32*	This work
MI301	*h- pmk1::ura4 pmk1-GFP:leu1+ ura4-D18 leu1-32*	J. Cansado's Lab
RD 5201	*h- pmk1::ura4+ pmk1-GFP:leu1+ slp1-HA:KanMX nda3-KM311*	This work
RD 5743	*h- pek1::ura4+ pmk1::ura4+ pmk1-GFP:leu1+ slp1-HA:KanMX nda3-KM311*	This work
RD 5525	*h+ pmk1::ura4+ pmk1-GFP:leu1+ slp1-MAPK Docking site-HA:KanMX nda3-KM311*	This work
RD 5511	*h+ pmk1::ura4+ slp1-MAPK Docking site-HA: KanMX ura4-D18 leu1-32*	This work
RD 5505	*h- slp1-MAPK Docking site-HA:KanMX nda3-KM311*	This work
RD 6189	*h mad2-GFP:KanMX slp1-MAPK Docking site-HA: KanMX nda3-KM311*	This work
RD 6083	*h slp1-MAPK Docking site-HA:KanMX mad3-GFP:his+ nda3-KM311 ura4-D18 leu1-32*	This work
TK107	h- leu 1–32 ura4-D18 sty1+:: ura4+	T. Kato's Lab
TK108	h- his1–102 leu 1–32 ura4-D18 wis1+::his1+	T. Kato's Lab
RD 6195	*h- pmk1::NatMX sty1::ura4+ slp1-HA:KanMX nda3-KM311*	This work
MI200	h^{+} *pmk1-HA6H:ura4*$^{+}$ *ura4D-18 leu1-32 ade6-M216*	J. Cansado's Lab
