# Supplementary material for: MAPK-dependent control of mitotic progression in S. pombe
Source: BMC Biol. 2024 Mar 25;22:71. doi: 10.1186/s12915-024-01865-6 (PMC10962199; doi:10.1186/s12915-024-01865-6)
Supplement: Supplementary file 4 — Additional file 4. Original blots used in this study. [file 12915_2024_1865_MOESM4_ESM.pdf]

Figure 2 .

Figure 2 A.

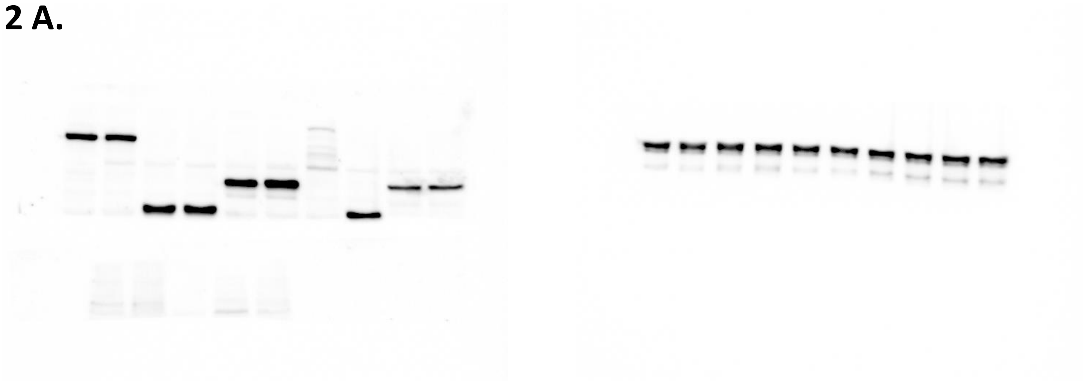

Figure 2 B.

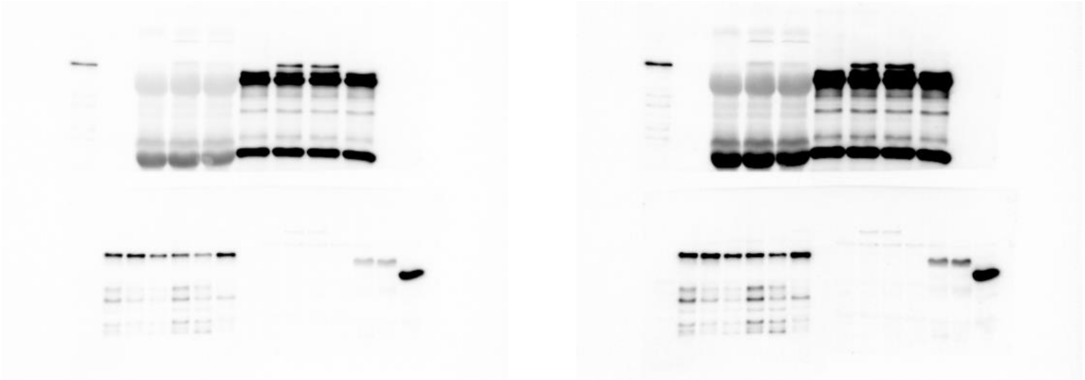

Figure 2 C.

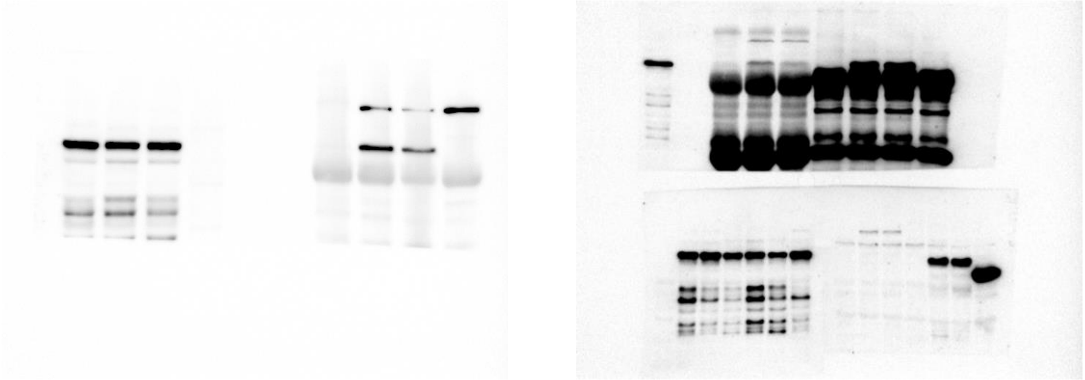

Figure 2 D.

Left.

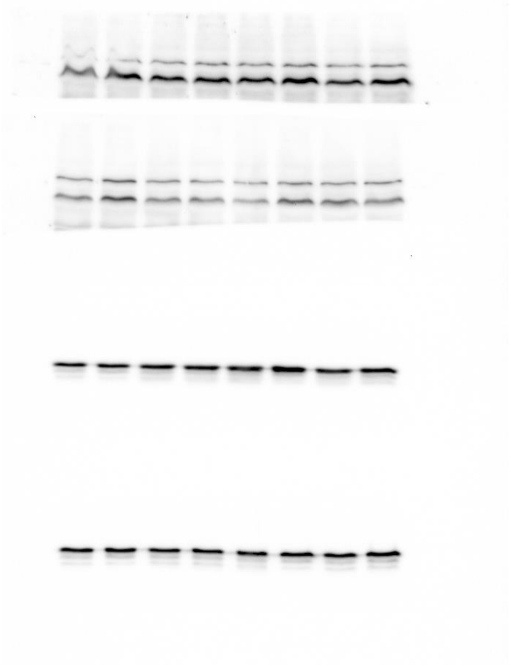

Right

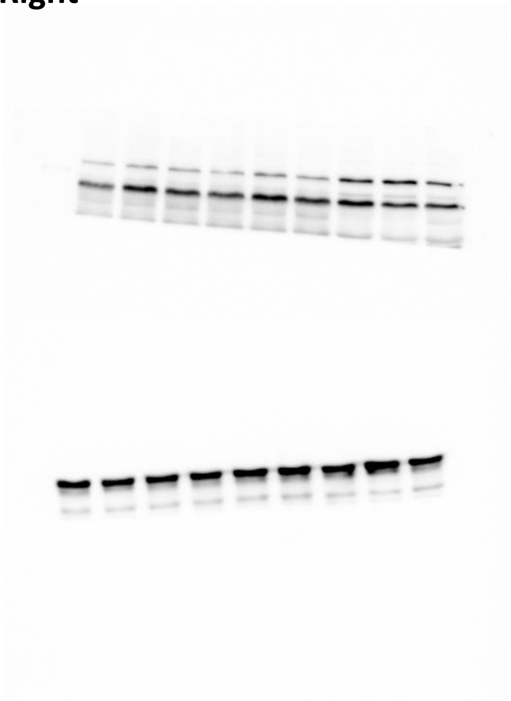

Figure 2 F

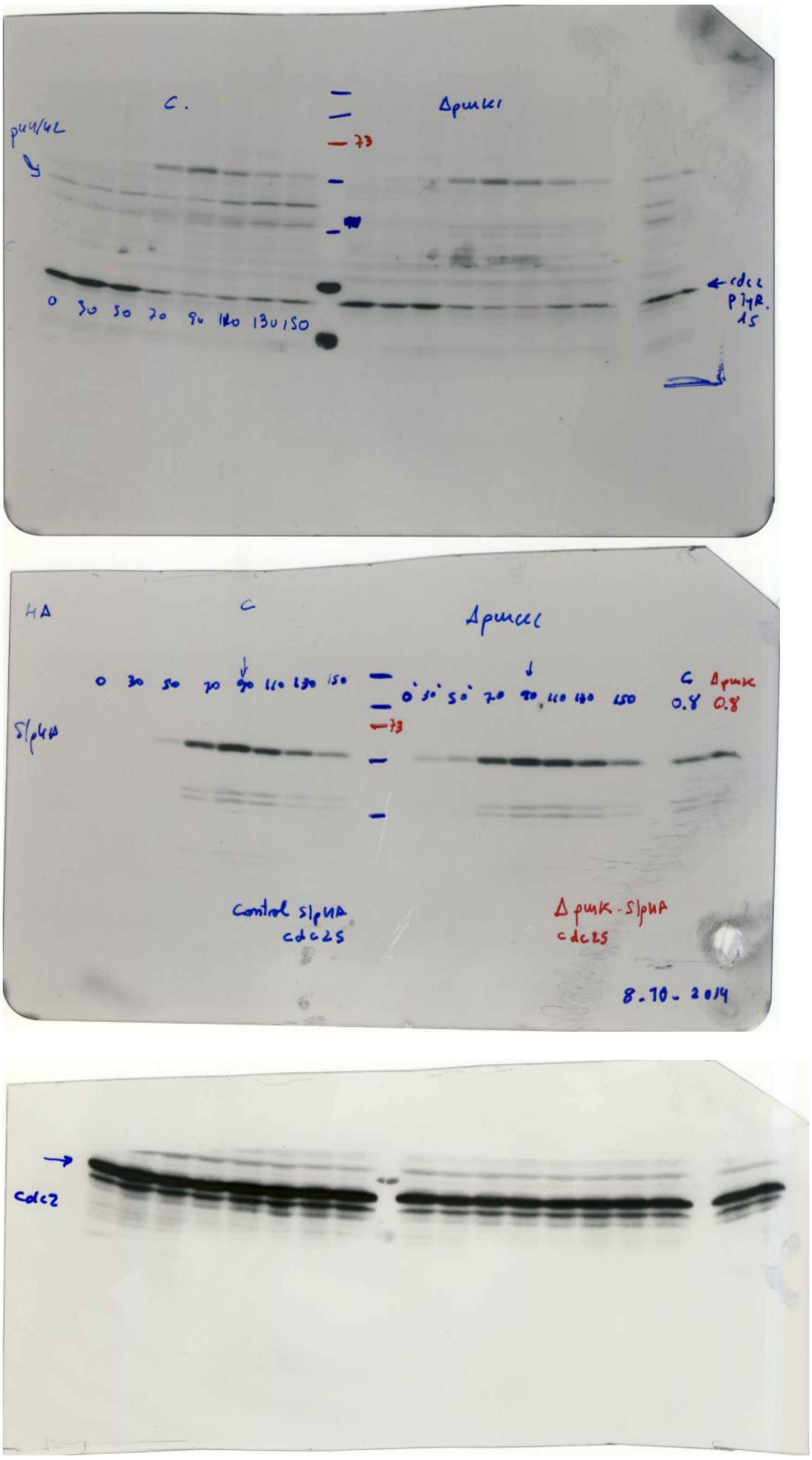

Figure 2 G.

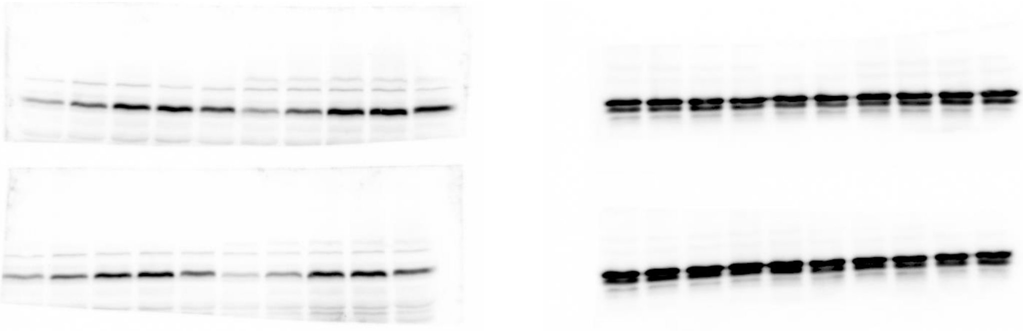

Figure 3 .

Figure 3 A.

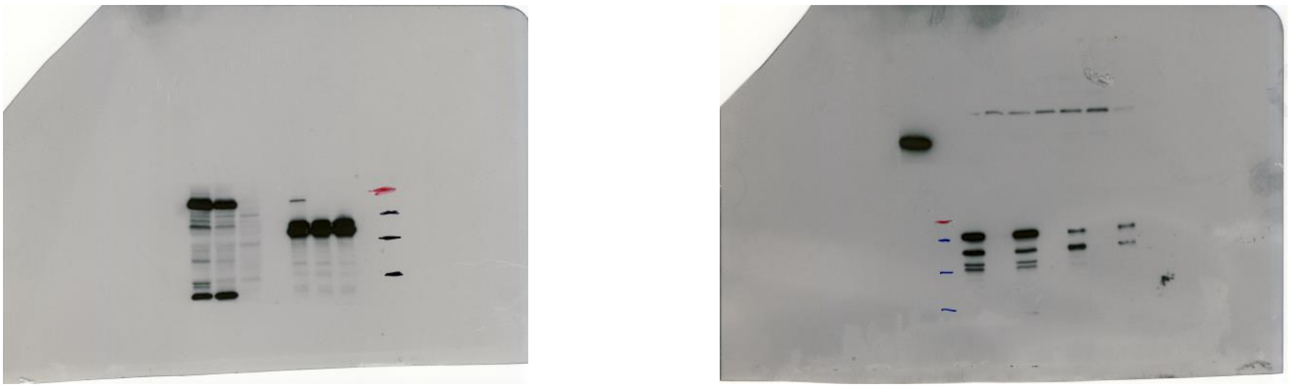

Figure 3 C.

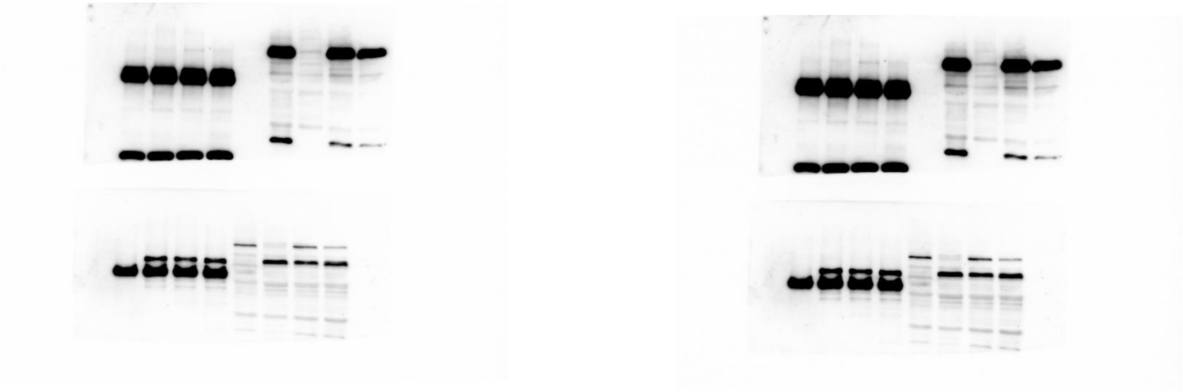

Figure 3 D.

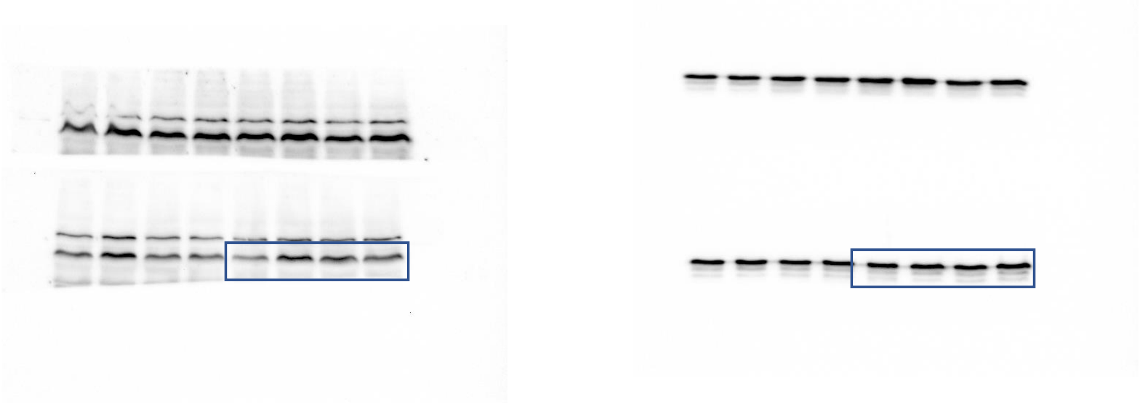

Figure 3 F.

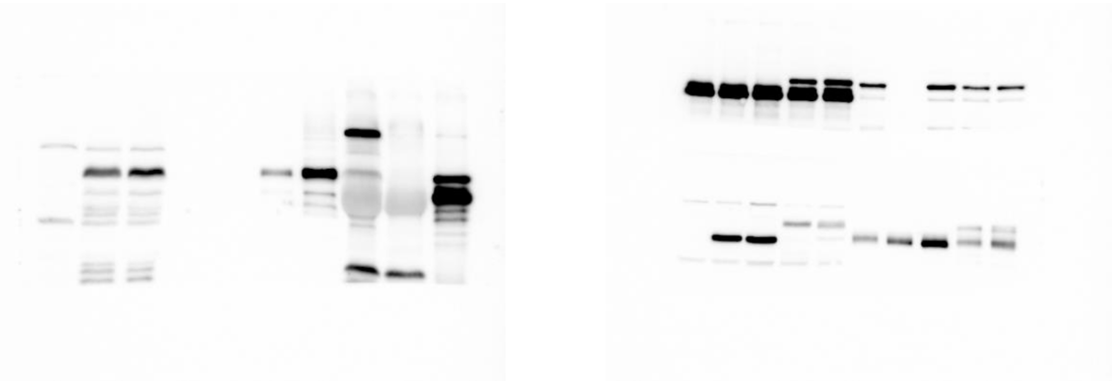

Figure 4 .

Figure 4 A. Left.

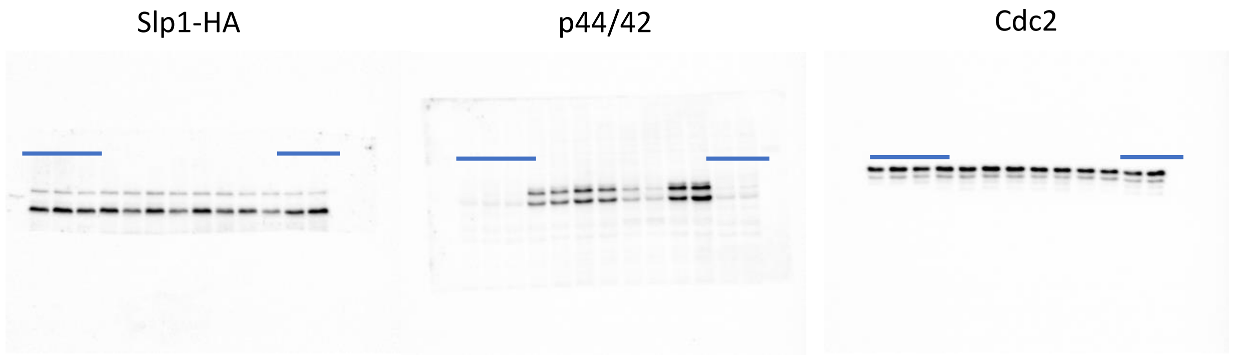

Figure 4 A. Right.

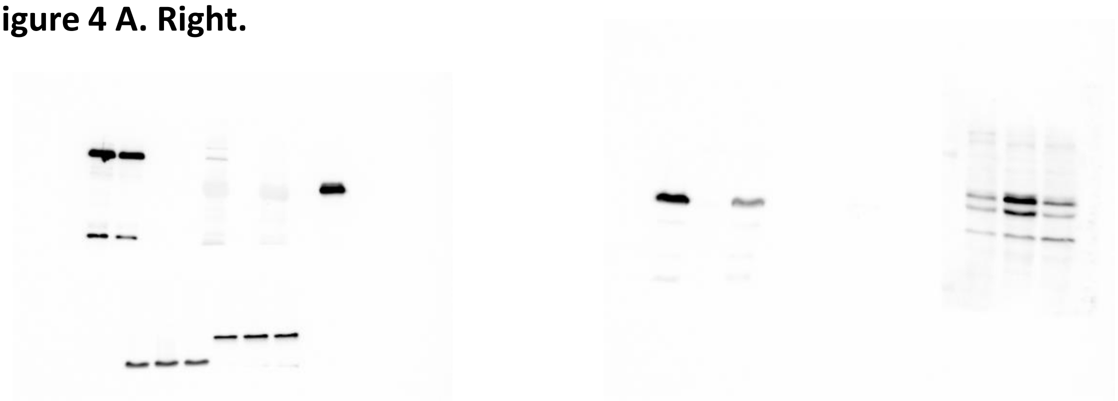

Figure 4 B

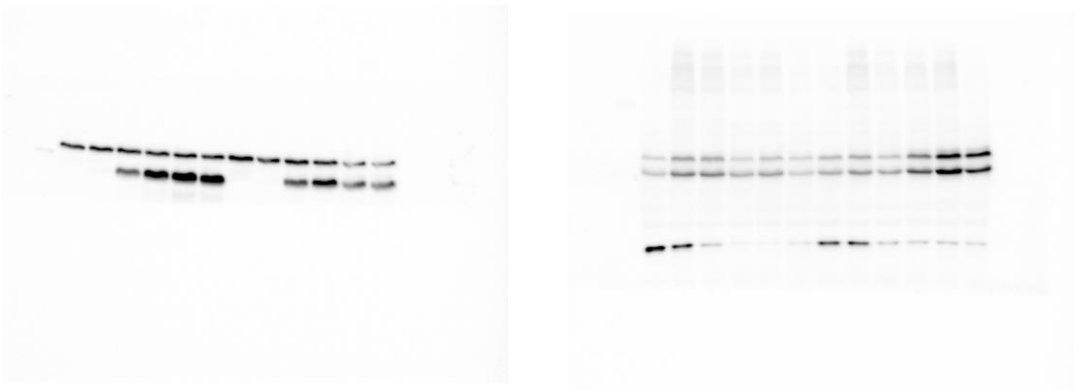

Figure 4 C

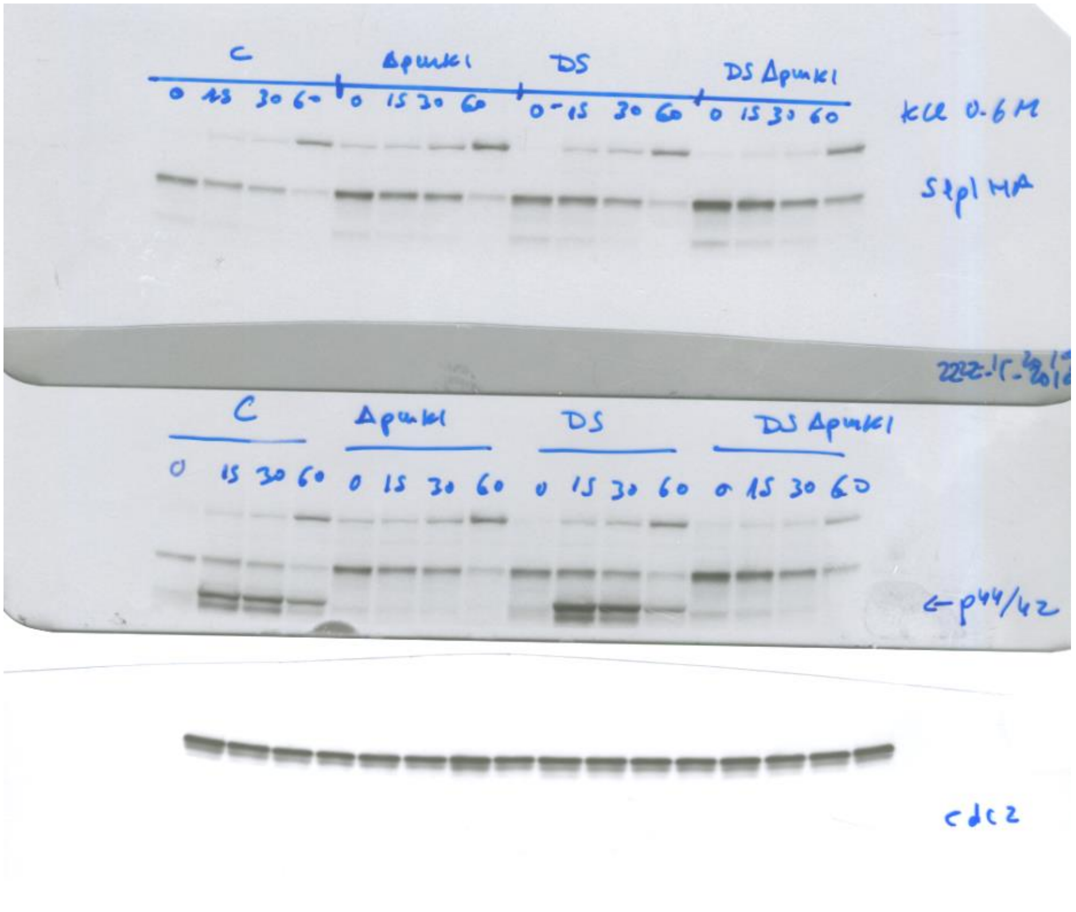

Figure 4 D

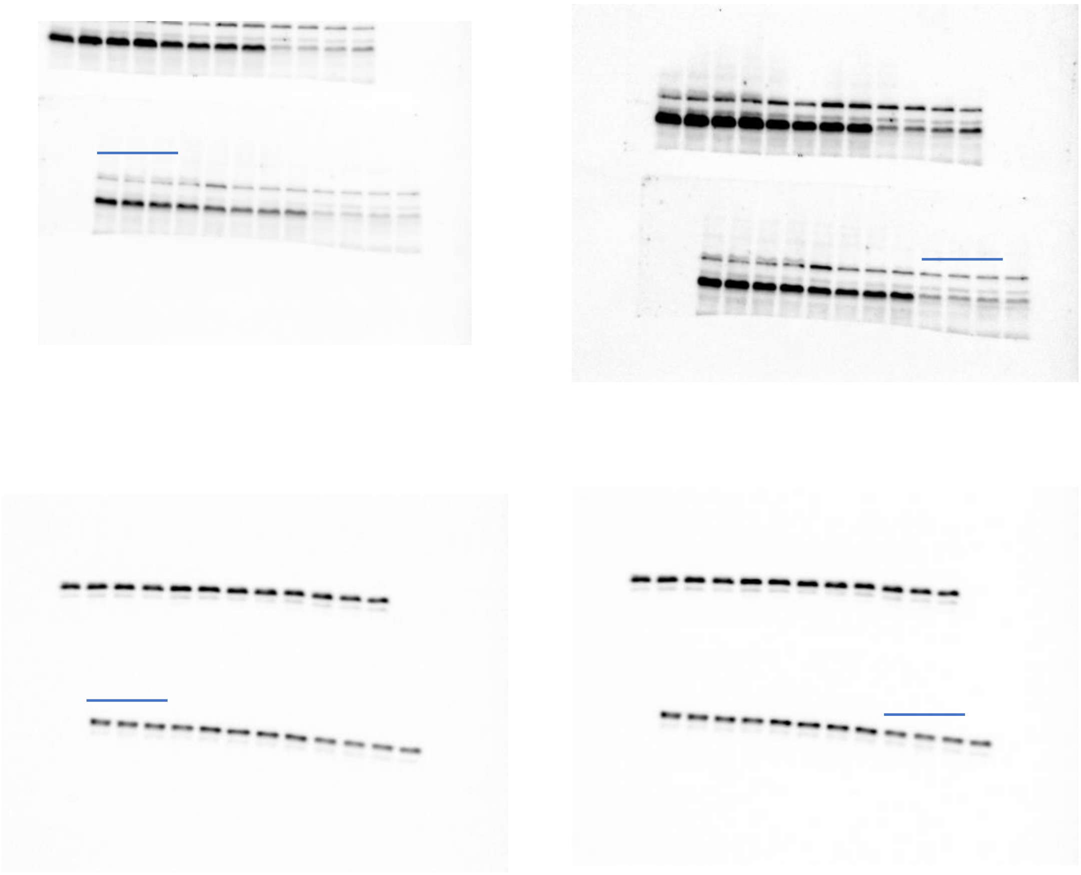

Figure 5 .

Figure 5 A.

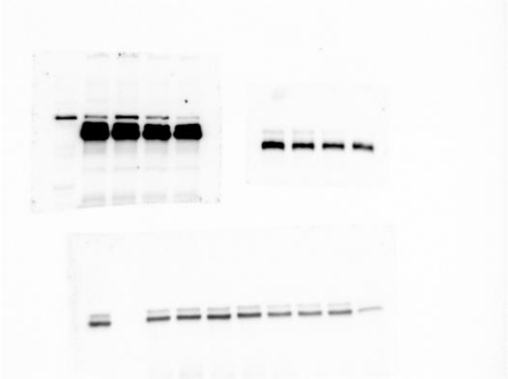

Figure 5 B.

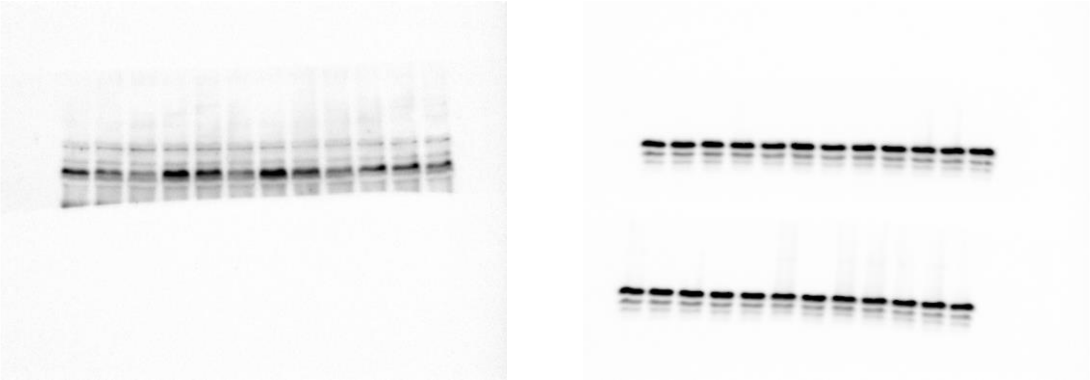

Figure 5 C.

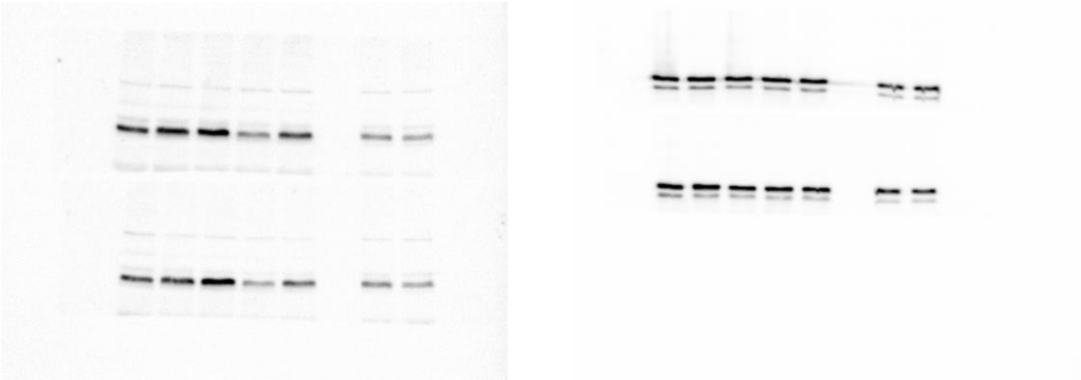

Figure 5 D.

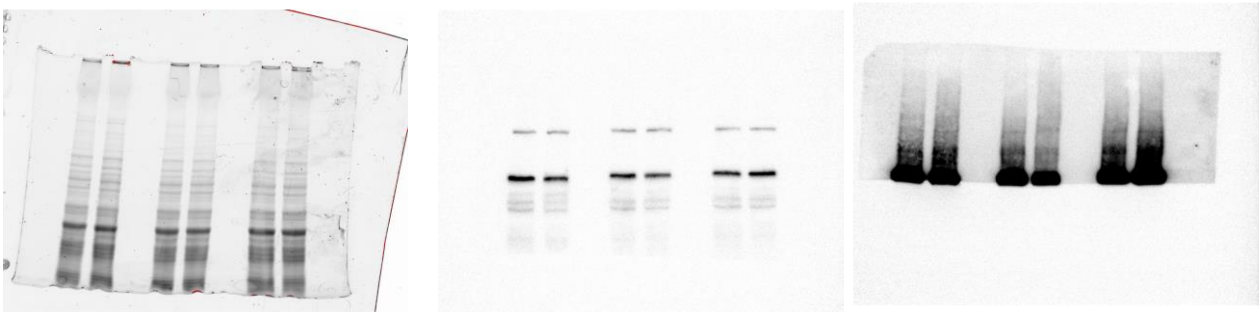

Figure S2 .

Figure S2 A.

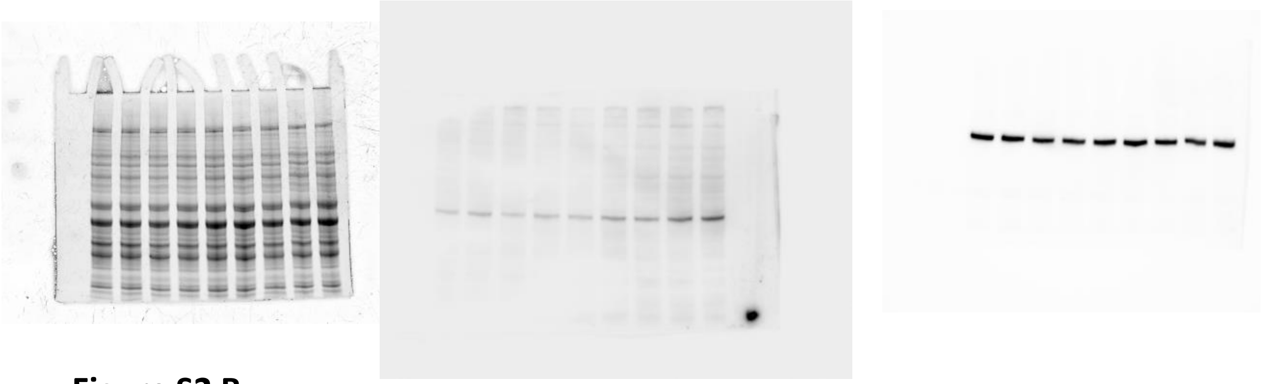

Figure S2 B.

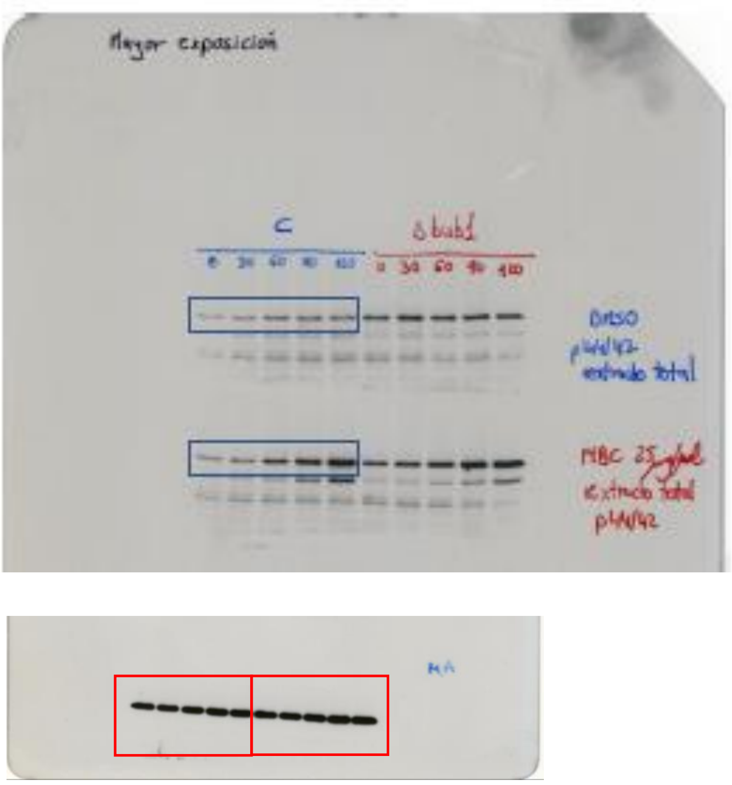

MBC (25µg/µl) Pmk1HA   Control DMSO Pmk1HA
